# Supplementary material for: Evaluating BindCraft for Generative Design of High-Affinity Peptides
Source: ACS Chem Biol. 2025 Nov 18;20(12):2991–8. doi: 10.1021/acschembio.5c00774 (PMC12723675; doi:10.1021/acschembio.5c00774)

# Supporting Information for:

# Evaluating BindCraft for Generative Design of High-Affinity Peptides

Mike Filius[1,2], Thanasis Patsos[1,2], Hugo Minee[1,2], Gianluca Turco[1,2], Henrick E. Chong[1,2], Jingming Liu[1,2], Monika Gnatzy[1,2], Ramon S.J. Rooth[1], Andy C. H. Liu[1], Rosa D.T. Ta[1], Isa H. A. Rijk[1], Safiya Ziani[1], Femke J. Boxman[1], and Sebastian J. Pomplun*[1,2]

*Corresponding author: s.j.pomplun@lacdr.leidenuniv.nl


| | |
|---|---|
| Table S1 | BLI screening for MDM2 binders at single concentration. |
| Figure S1 | Competition assay for MDM2 with binders B1 and B17 and binding curve for p53-MDM2 interaction |
| Figure S2 | Crystal structures for WDR5 in complex with the MLL and Myc binding peptide |
| Table S2 | BLI screening for WDR5 WIN binders at single concentration |
| Table S3 | BLI screening for WDR5 Myc binders at single concentration |
| Figure S3 | BLI analysis for the WDR5_Myc B1 scrambled sequence |
| Figure S4 | Competition assay for WDR5 with binders B1 and B1 stapled. |
| Figure S5 | WDR5 WIN peptide stapling and association curves |
| Figure S6 | Predicted PD1/PD-L1 Peptide Binders and BLI Binding Analysis. |
| Table S4 | Overview of BindCraft input settings used for different protein targets. |
| Table S5 | WDR5 Myc B1 stapled sequence. |
| Figures S6-21 | LC-MS Analysis for MDM2 peptides |
| Figures S22-34 | LC-MS Analysis for WDR5 peptides |
| Figure S35 | LC-MS Analysis for Myc-peptide-motif for WDR5 competition assay |
| Figures S36 | LC-MS Analysis for p53-peptide-motif for MDM2 competition assay |
| Figures S37 and 38 | LC-MS Analysis for MDM2 B1 and B17 used for competition assay |
| Figure S39 | LC-MS Analysis for WDR5_Myc_B1 used for competition assay |
| Figures S40 and 41 | LC-MS Analysis for WDR5_Myc_B1 used for stapling and competition assay |
| Figures S42-52 | LC-MS Analysis for PD1/PDL1 peptides |

**Table S1:** BLI screening for MDM2 binders at 1 µM of MDM2.

| Binder | KD (M) | KD Error | ka (1/Ms) | ka Error | kdis (1/s) | kdis Error | Full R^2 |
|---|---|---|---|---|---|---|---|
| MDM2_B1 | 3.428E-07 | 1.051E-07 | 1.141E04 | 2.459E03 | 3.913E-03 | 8.534E-04 | 0.8456 |
| MDM2_B11 | 2.500E-07 | 4.357E-08 | 3.542E04 | 4.051E03 | 8.856E-03 | 1.164E-03 | 0.9237 |
| MDM2_B12 | 2.756E-07 | 6.971E-08 | 1.672E04 | 2.920E03 | 4.609E-03 | 8.432E-04 | 0.8588 |
| MDM2_B13 | 2.044E-07 | 3.998E-08 | 2.994E04 | 4.196E03 | 6.119E-03 | 8.348E-04 | 0.8879 |
| MDM2_B14 | 4.956E-07 | 1.713E-07 | 8.154E03 | 2.008E03 | 4.041E-03 | 9.801E-04 | 0.8403 |
| MDM2_B16 | 4.331E-07 | 3.445E-08 | 1.801E04 | 7.948E02 | 7.800E-03 | 5.162E-04 | 0.9853 |
| MDM2_B17 | 3.044E-07 | 2.701E-08 | 1.873E04 | 1.115E03 | 5.702E-03 | 3.753E-04 | 0.9729 |
| MDM2_B18 | 3.340E-07 | 8.472E-08 | 1.439E04 | 2.557E03 | 4.806E-03 | 8.696E-04 | 0.8752 |
| MDM2_B2 | 6.174E-07 | 1.581E-07 | 9.642E03 | 1.757E03 | 5.953E-03 | 1.071E-03 | 0.9006 |
| MDM2_B20 | 7.359E-07 | 1.052E-07 | 1.316E04 | 9.160E02 | 9.687E-03 | 1.210E-03 | 0.9763 |
| MDM2_B3 | 1.175E-06 | 1.781E-07 | 8.517E03 | 1.926E02 | 1.000E-02 | 1.500E-03 | 0.9932 |
| MDM2_B4 | 9.647E-07 | 1.048E-07 | 9.395E03 | 2.304E02 | 9.064E-03 | 9.589E-04 | 0.9913 |
| MDM2_B5 | 8.376E-07 | 1.249E-07 | 1.165E04 | 2.062E02 | 9.757E-03 | 1.444E-03 | 0.9901 |
| MDM2_B6 | 2.639E-06 | 5.577E-08 | 9.427E03 | 5.397E01 | 2.488E-02 | 5.061E-04 | 0.9962 |
| MDM2_B8 | 7.830E-07 | 1.598E-07 | 8.044E03 | 9.689E02 | 6.299E-03 | 1.038E-03 | 0.9296 |

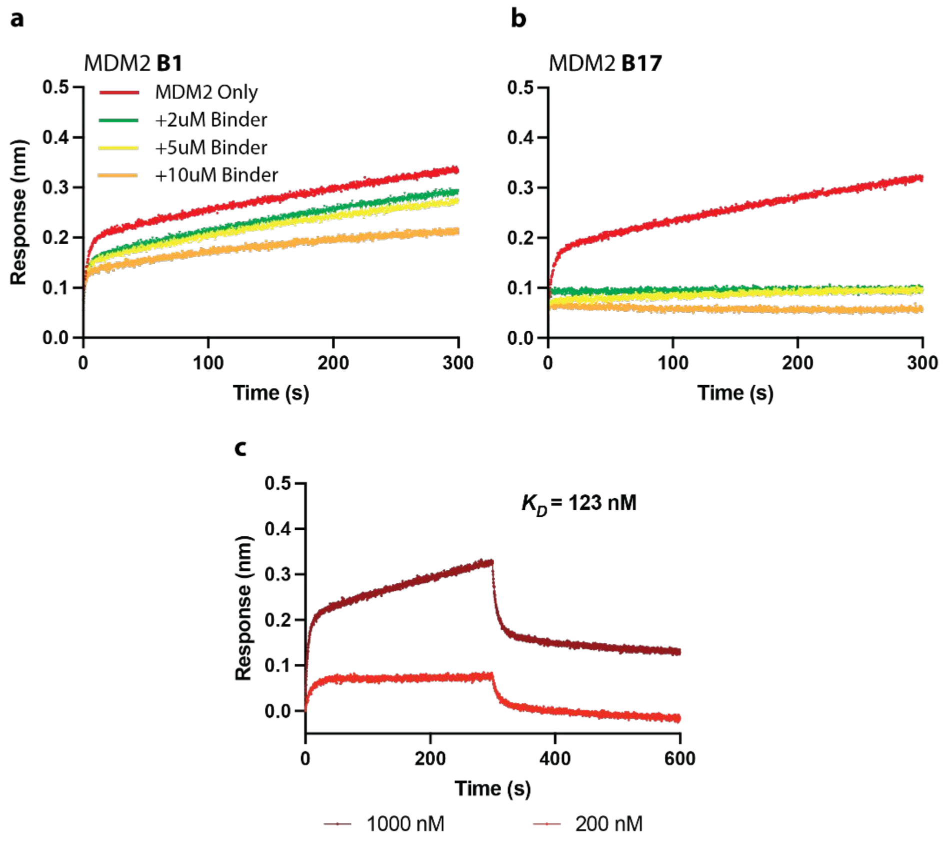


**Figure S1: BLI analysis for MDM2 competition assay and p53 binding curve. a and b)** Competition analysis (BLI association) of two predicted binders (B1 and B17) to MDM2-p53. P53 immobilized on BLI tips was dipped into solutions containing 1 µM MDM2 and different concentrations of the binders (2, 5, and 10 µM). Increasing the concentration of the predicted binders (B1 and B17) in solution causes occupancy of the MDM2-p53 binding pocket and results in a concentration-dependent decrease in BLI response. **c**) Full binding curves for p53 and MDM2, with dissociation constants ($K_D$) calculated as the average from four different concentrations (1000 and 200 nM).

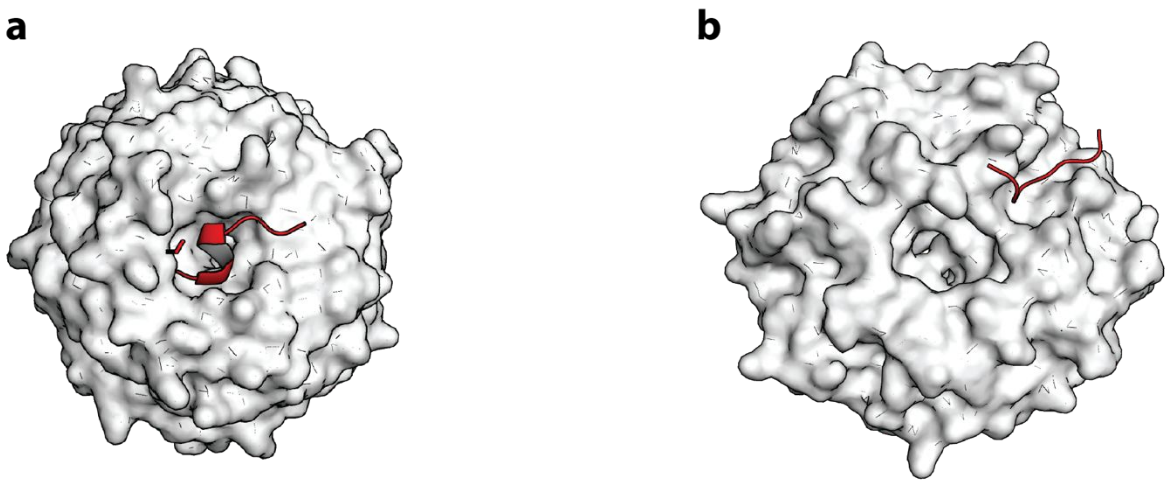


**Figure S2:** Crystal structures for WDR5 in complex with the MLL binding motif (panel a, PDB: 3UVM) and Myc binding motif (panel b, PDB: 4Y7R). peptides are highlighted in red, WDR5 crystal structure in white.

**Supplementary Table 2:** BLI screening for WDR5 WIN binders at 2 µM of WDR5.

| Sample ID | Response | KD (M) | KD Error | ka (1/Ms) | ka Error | kdis (1/s) | kdis Error |
|---|---|---|---|---|---|---|---|
| Win_B1 | -1.780E-02 | 1.000E-07 | 0.000E00 | 1.000E04 | 0.000E00 | 1.000E-03 | 0.000E00 |
| Win_B8 | -3.945E-02 | 1.000E-07 | 0.000E00 | 1.000E04 | 0.000E00 | 1.000E-03 | 0.000E00 |
| Win_B2 | -1.082E-02 | 1.000E-07 | 0.000E00 | 1.000E04 | 0.000E00 | 1.000E-03 | 0.000E00 |
| Win_B9 | -4.241E-02 | 1.000E-07 | 0.000E00 | 1.000E04 | 0.000E00 | 1.000E-03 | 0.000E00 |
| Win_B5 | -3.939E-02 | 1.000E-07 | 0.000E00 | 1.000E04 | 0.000E00 | 1.000E-03 | 0.000E00 |
| Win_B10 | -3.857E-02 | 1.000E-07 | 0.000E00 | 1.000E04 | 0.000E00 | 1.000E-03 | 0.000E00 |
| Win_B6 | -4.174E-02 | 1.000E-07 | 0.000E00 | 1.000E04 | 0.000E00 | 1.000E-03 | 0.000E00 |
| Win_B11 | -2.580E-02 | 1.000E-07 | 0.000E00 | 1.000E04 | 0.000E00 | 1.000E-03 | 0.000E00 |

**Table S3:** BLI screening for WDR5 Myc binders at 2 µM of WDR5.

| Sample ID | Response | KD (M) | KD Error | ka (1/Ms) | ka Error | kdis (1/s) | kdis Error | Full R^2 |
|---|---|---|---|---|---|---|---|---|
| Myc_B1 | 1.2214 | 4.396E-08 | 6.287E-10 | 8.282E03 | 3.012E01 | 3.641E-04 | 5.036E-06 | 0.9858 |
| Myc_B8 | 0.8834 | 1.045E-07 | 1.567E-09 | 6.985E03 | 4.794E01 | 7.303E-04 | 9.731E-06 | 0.9532 |
| Myc_B3 | -9.846E-03 | 1.000E-07 | 0.000E00 | 1.000E04 | 0.000E00 | 1.000E-03 | 0.000E00 | |
| Myc_B10 | 0.3503 | 2.040E-07 | 2.815E-09 | 9.039E03 | 9.548E01 | 1.844E-03 | 1.638E-05 | 0.902 |
| Myc_B5 | 0.0129 | 2.598E-04 | 4.592E-02 | 1.930E03 | 3.411E05 | 5.014E-01 | 4.768E-01 | 0.1212 |
| Myc_B11 | 0.3188 | 1.869E-07 | 2.367E-09 | 7.889E03 | 6.984E01 | 1.475E-03 | 1.335E-05 | 0.9241 |
| Myc_B6 | 1.3049 | 9.236E-08 | 1.278E-09 | 6.037E03 | 3.147E01 | 5.576E-04 | 7.150E-06 | 0.9779 |
| Myc_B12 | -1.519E-02 | 1.000E-07 | 0.000E00 | 1.000E04 | 0.000E00 | 1.000E-03 | 0.000E00 | |
| Myc_B7 | 0.4711 | 1.455E-07 | 1.938E-09 | 1.068E04 | 1.030E02 | 1.553E-03 | 1.427E-05 | 0.9054 |

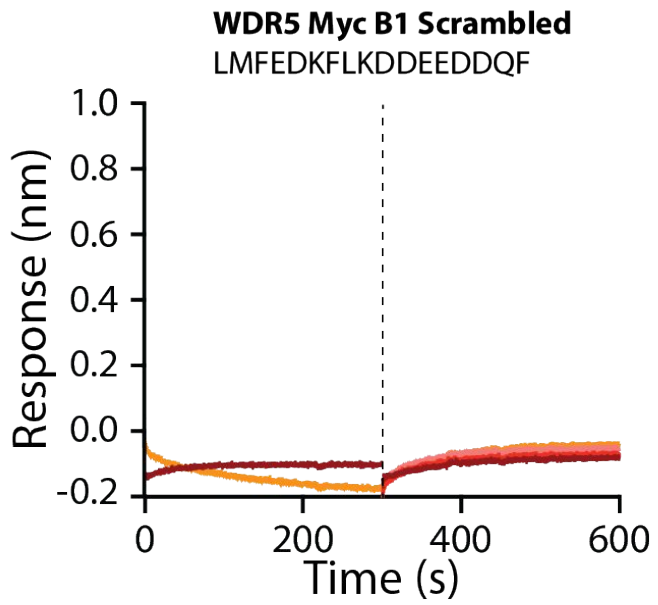


**Figure S3: BLI analysis for the WDR5_Myc B1 scrambled sequence.** BLI affinity measurement for a scrambled version of the WDR5_Myc_B1 peptide. Biotinylated scrambled peptide (100 nM) was loaded onto BLI tips, and responses were recorded for WDR5 at 5, 1, 0.2, and 0.04 µM. No binding was detected, confirming that the interaction observed for WDR5_Myc_B1 is sequence-specific rather than due to nonspecific electrostatic effects.

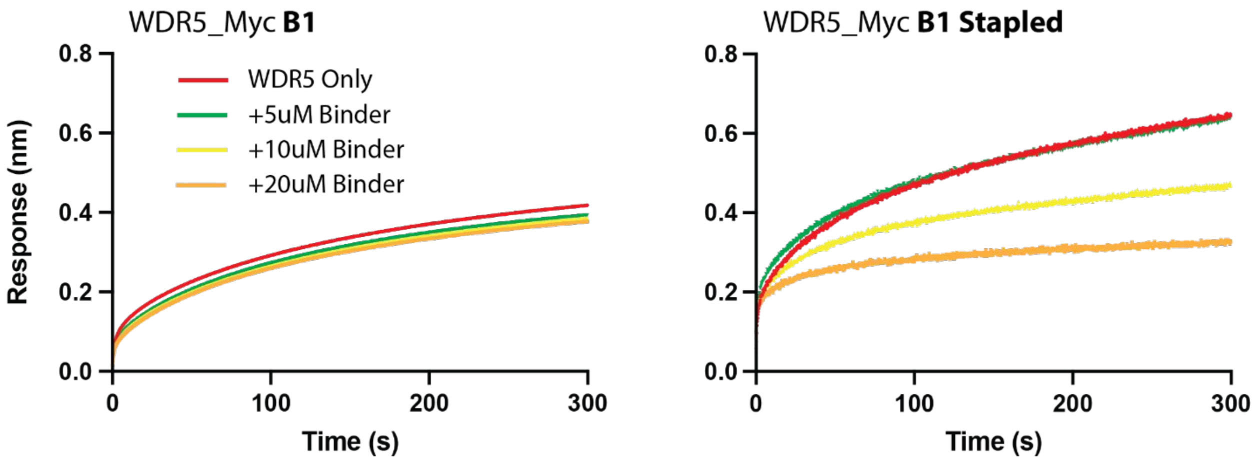


**Figure S4: Competition assay for WDR5 with binders B1 and B1 stapled.** Competition analysis (BLI association) of WDR5_Myc Binder1 vs its stapled variant to WDR5-Myc interaction. A Myc-peptide motif was immobilized on BLI tips and was dipped into solutions containing 2.5 µM WDR5 and different concentrations of the binders (5, 10, and 20 µM). Increasing the concentration of the binders in solution causes occupancy of the WDR5-Myc binding pocket and results in a concentration-dependent decrease in BLI response.

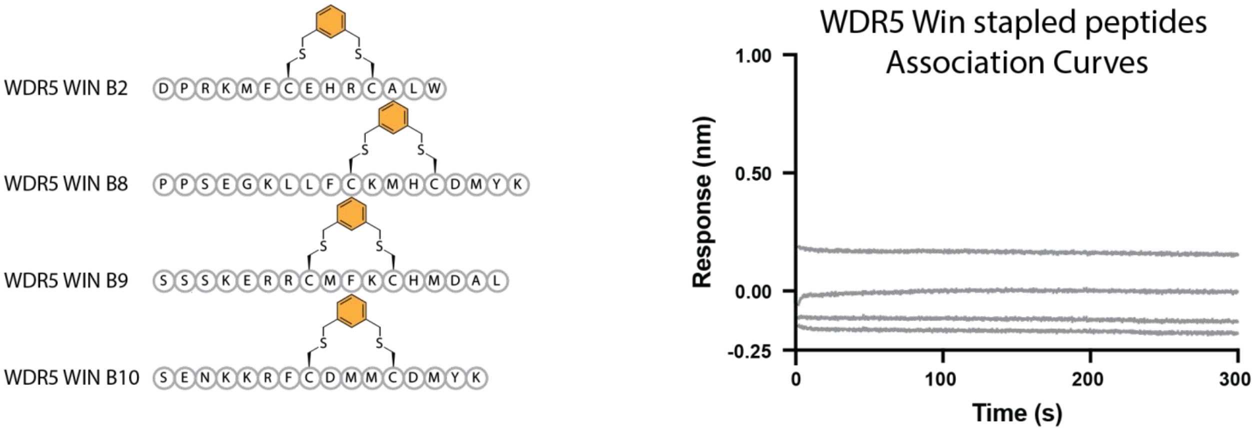


**Figure S5: WDR5 WIN peptide stapling and association curves.** Schematic representation of four selected WDR5–WIN peptide binders with cysteines introduced in an i, i+4 configuration and stapled using 1,3-bis(bromomethyl)benzene. The stapled peptides were immobilized on BLI tips and exposed to 2.5 µM WDR5. The association phase was measured; unfortunately, none of the stapled peptides produced a detectable binding signal.

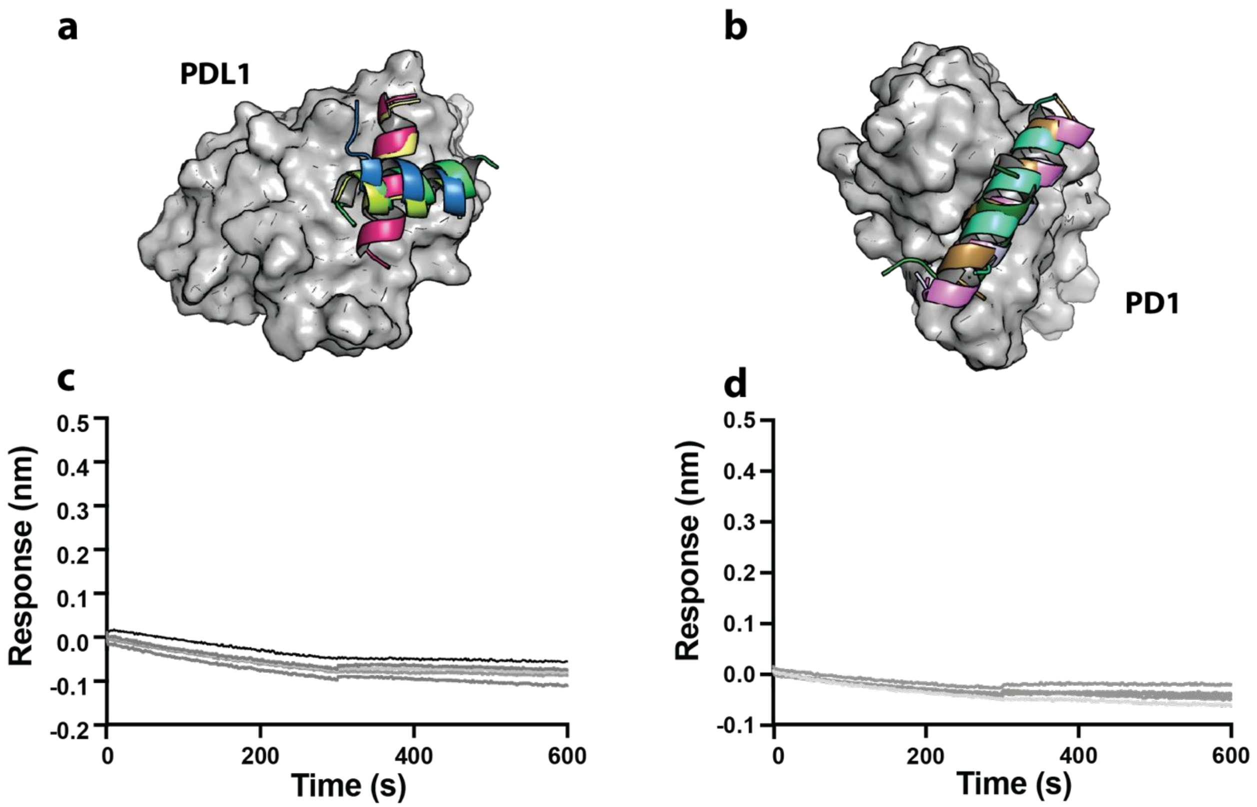


**Figure S6: Predicted PD1/PD-L1 Peptide Binders and BLI Binding Analysis.** Crystal structures for PDL1 (panel a) and PD1 (panel b) with predicted peptides targeting PDL1/PD1 interaction interface shown as colored helices. c and d) Binding affinities of the top 5 predicted peptides for PDL1 (panel c) and PD1 (panel d) assessed via BLI; none showed detectable binding with 1 μM of target protein.

**Table S4.** Overview of BindCraft input settings used for different protein targets.

| Target Protein | PDB | Target Hotspot | Peptide Length |
|---|---|---|---|
| MDM2 | 1YCR | 73-94 | 10-20 |
| WDR5_WIN | 6DY7 | 133 | 10-20 |
| WDR5_Myc | 6DY7 | 240 | 10-20 |
| PD1 | 4ZQK, chain B | 66 | 10-20 |
| PDL1 | 4ZQK, chain A | 111-127 | 10-20 |

**Table S5.** WDR5 Myc B1 stapled sequence.

| Peptide | Native sequence | Cys substitutions |
|---|---|---|
| WDR5_Myc_B1 | DDEDFEQFMKDLDEFLK | DDEDF**C**QFM**C**DLDEFLK |
| WDR5_Win_B2 | DPRKMFEEHRNALW | DPRKMF**C**EHR**C**ALW |
| WDR5_Win_B8 | PPSEGKLLFQKMHDDMYK | PPSEGKLLF**C**KMH**C**DMYK |
| WDR5_Win_B9 | SSSKERREMFKEHMDAL | SSSKERR**C**MFK**C**HMDAL |
| WDR5_Win_B10 | SENKKRFEDMMNDMYK | SENKKRF**C**DMM**C**DMYK |

**Figure S6: LC-MS Analysis MDM2_B1**

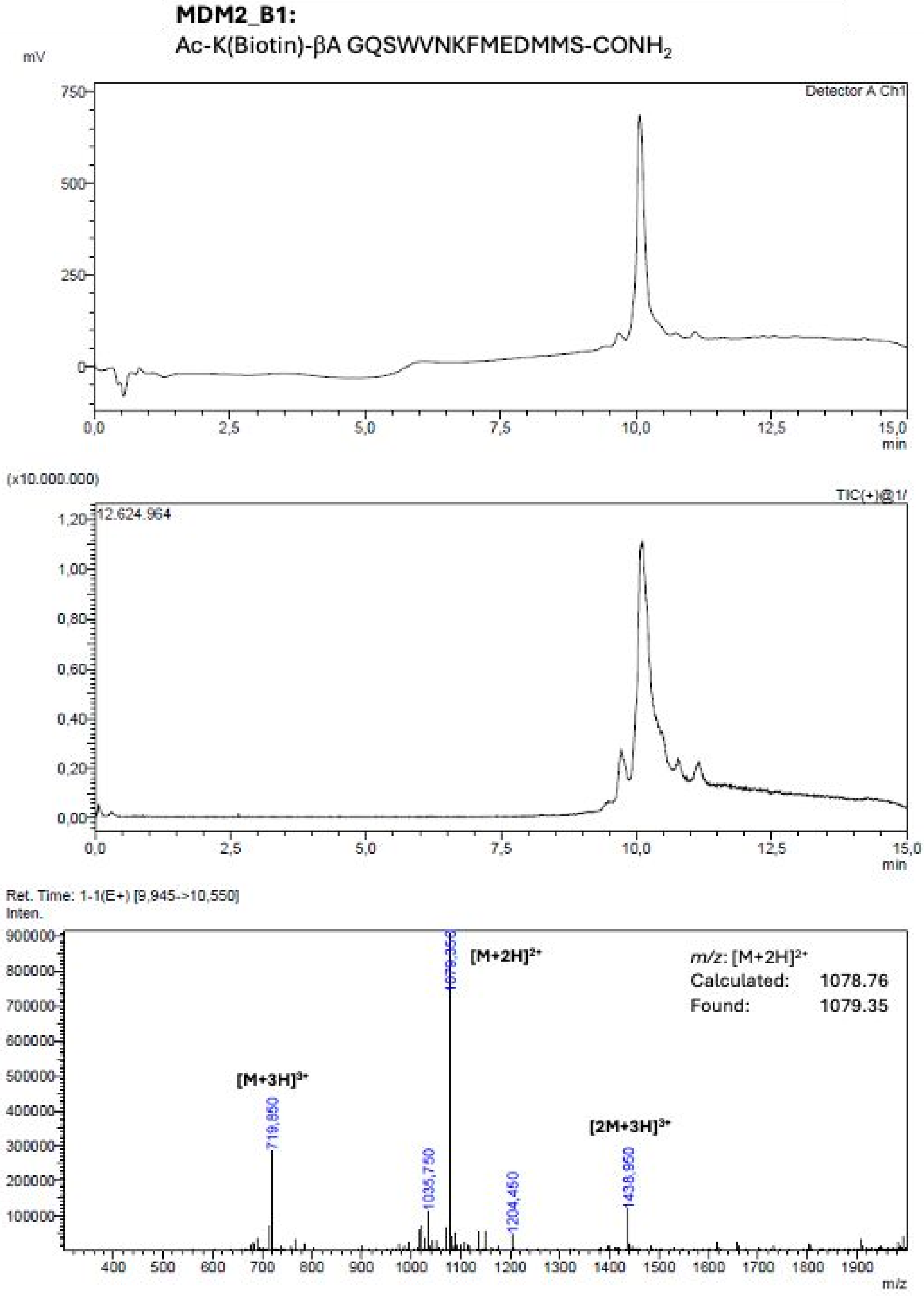

**Figure S7: LC-MS Analysis MDM2_B2**

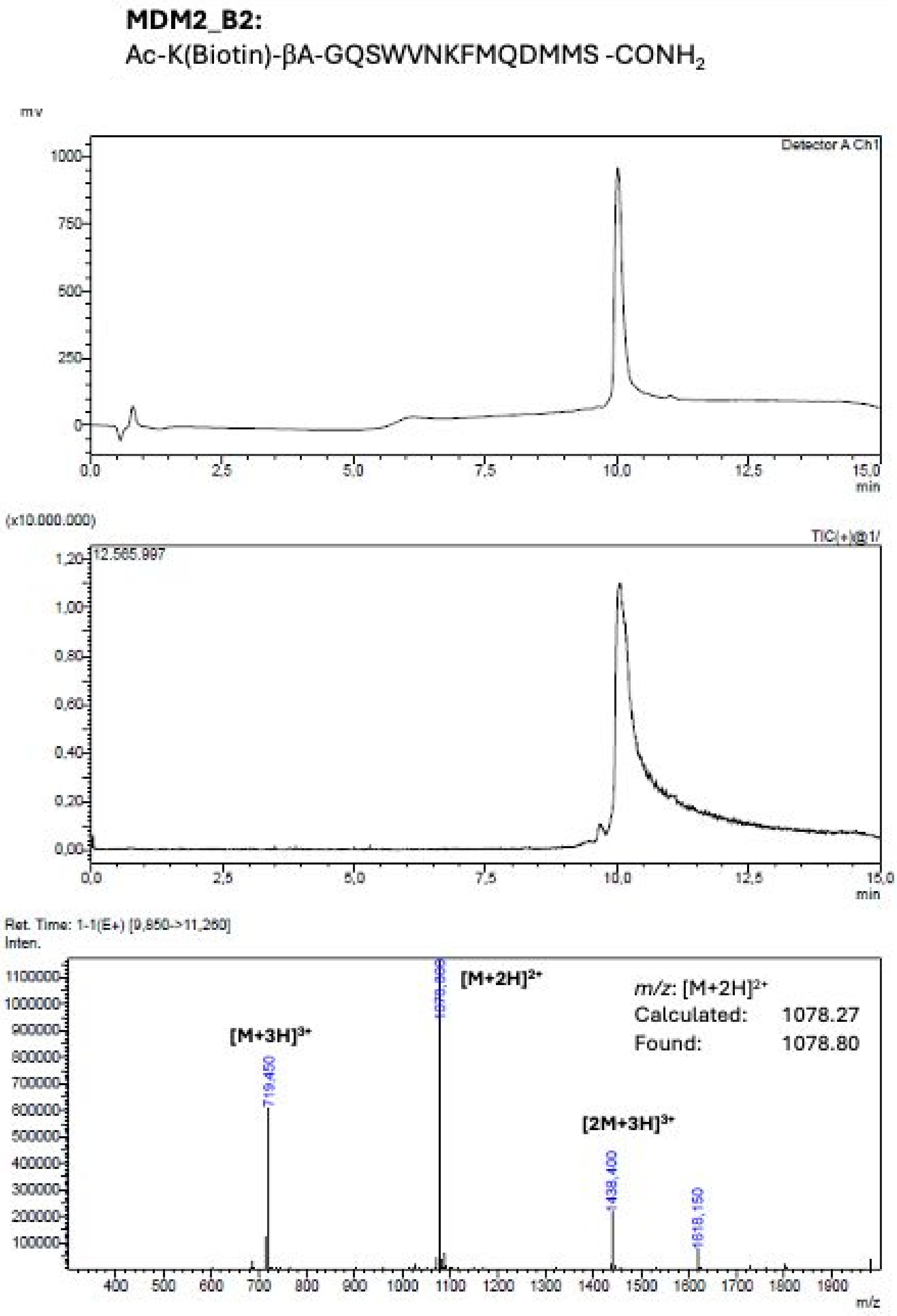

**Figure S8: LC-MS Analysis MDM2_B3**

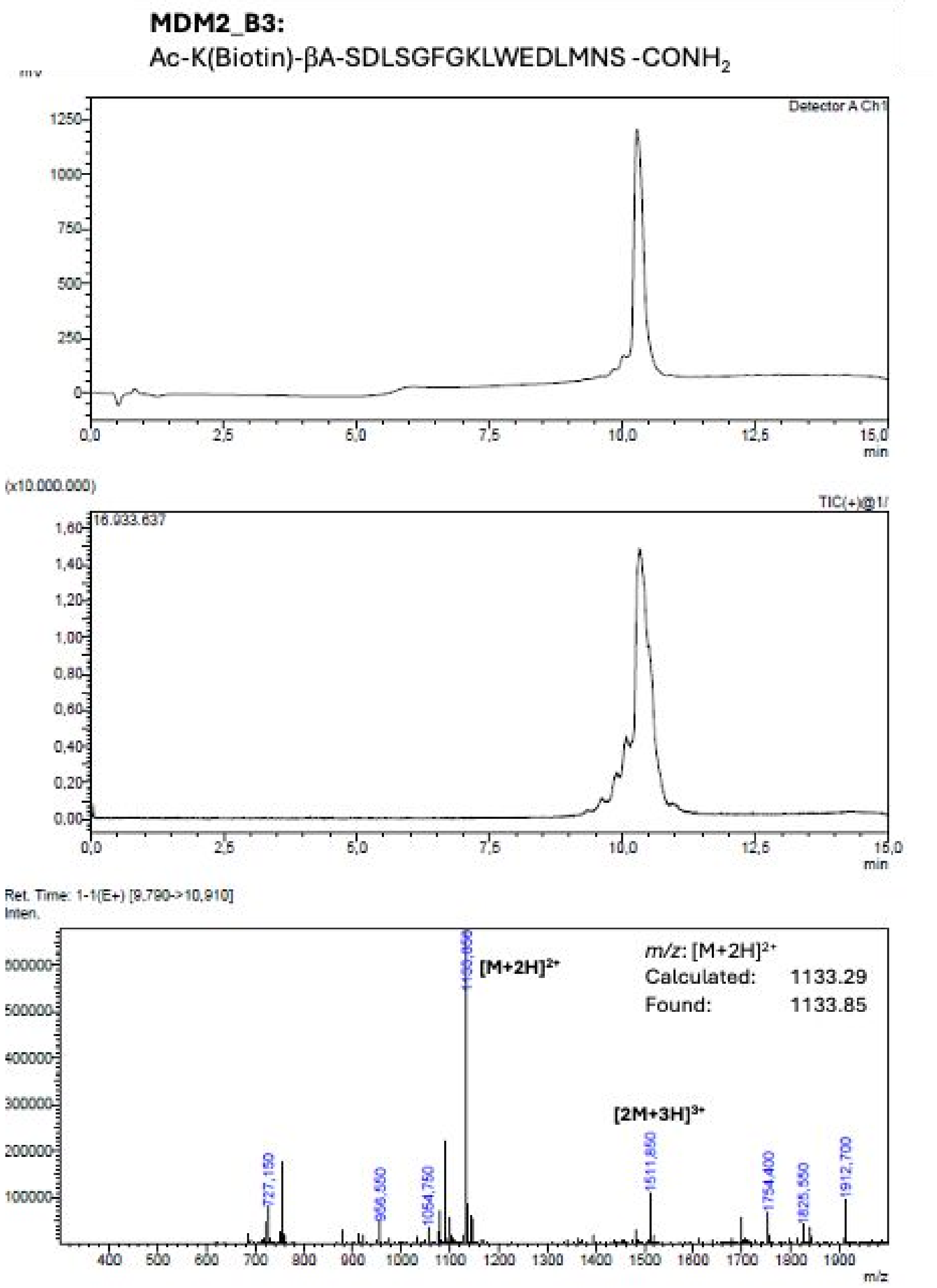

**Figure S9: LC-MS Analysis MDM2_B4**

**MDM2_B4:**
Ac-K(Biotin)-βA-SDLSGFGKLWQDLMNS -$CONH_2$

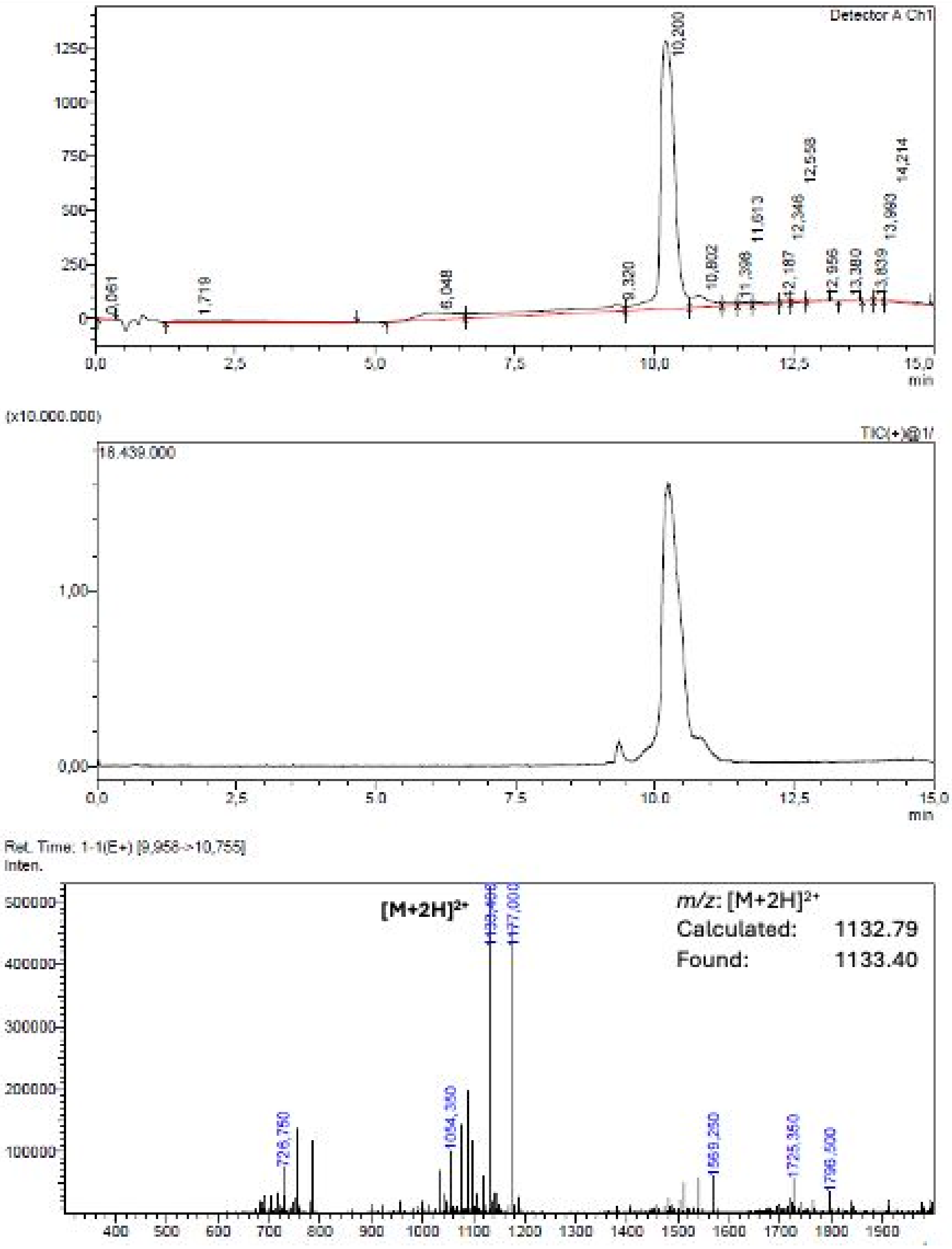

**Figure S10: LC-MS Analysis MDM2_B5**

**MDM2_B5:**

Ac-K(Biotin)-βA-SASDEFQKEWEDLMNF -$CONH_2$

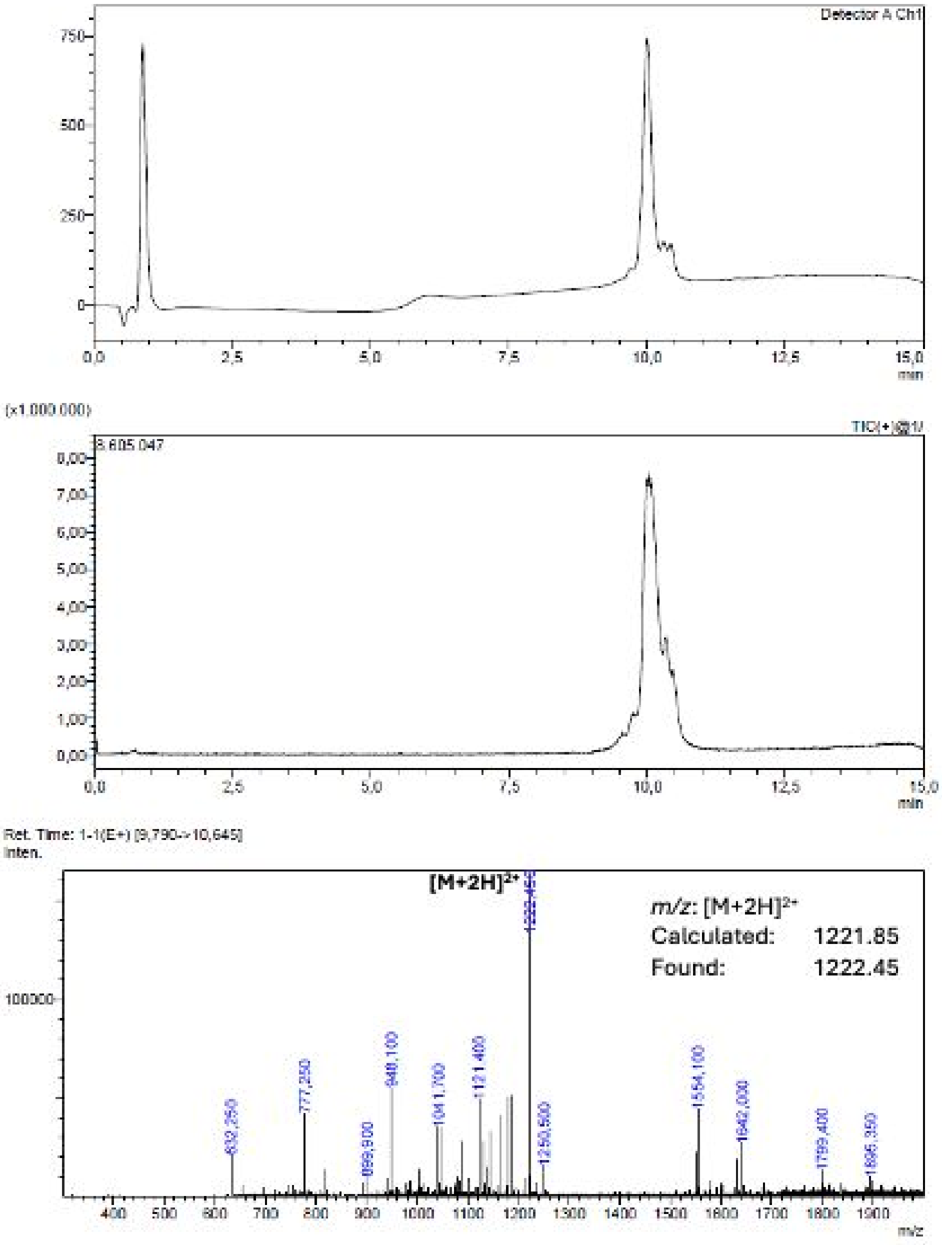

**Figure S11: LC-MS Analysis MDM2_B6**

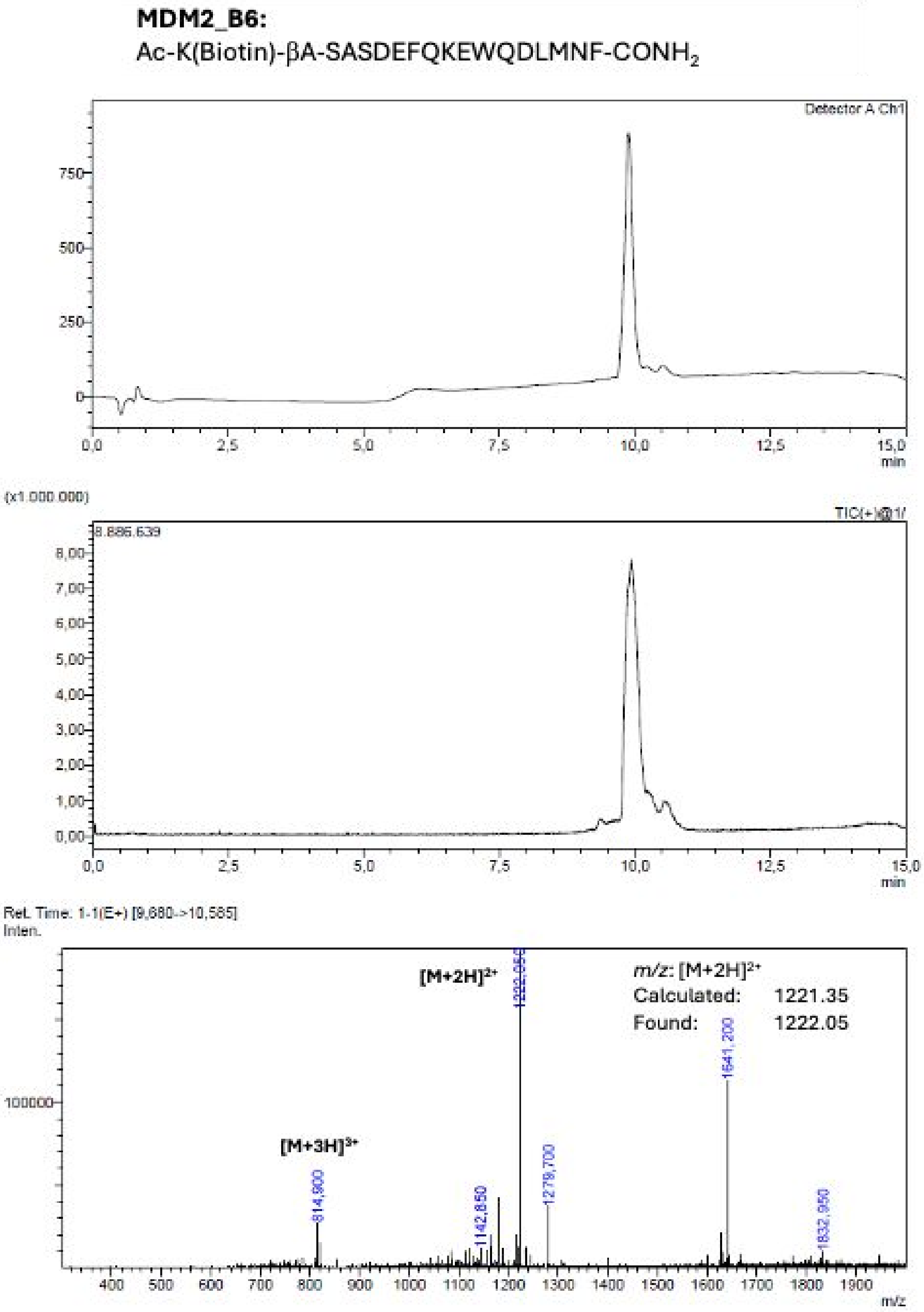

**Figure S12: LC-MS Analysis MDM2_B8**

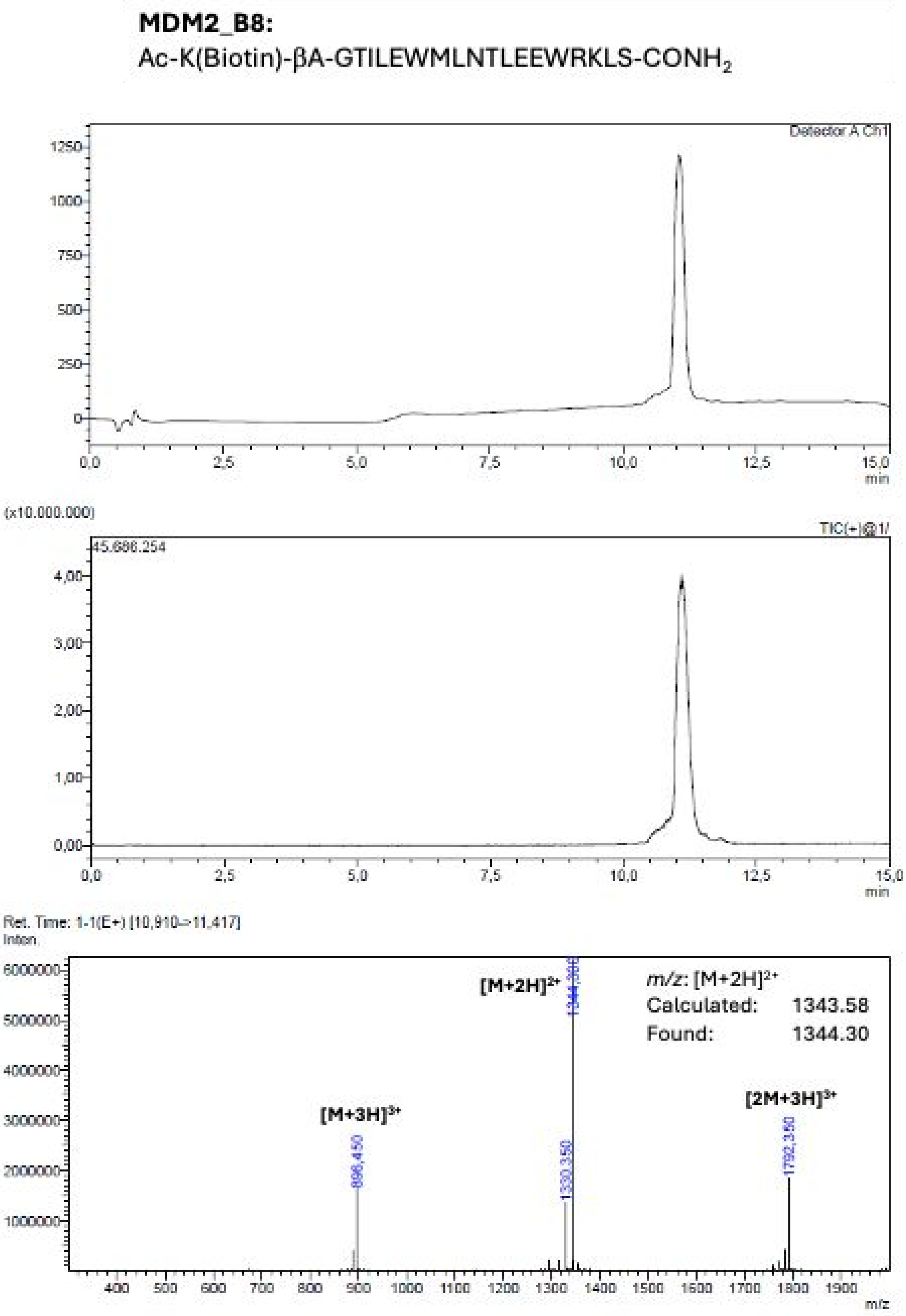

**Figure S13: LC-MS Analysis MDM2_B11**

**MDM2_B11:**
Ac-K(Biotin)-βA-TFSEQWQELLNS-$CONH_2$

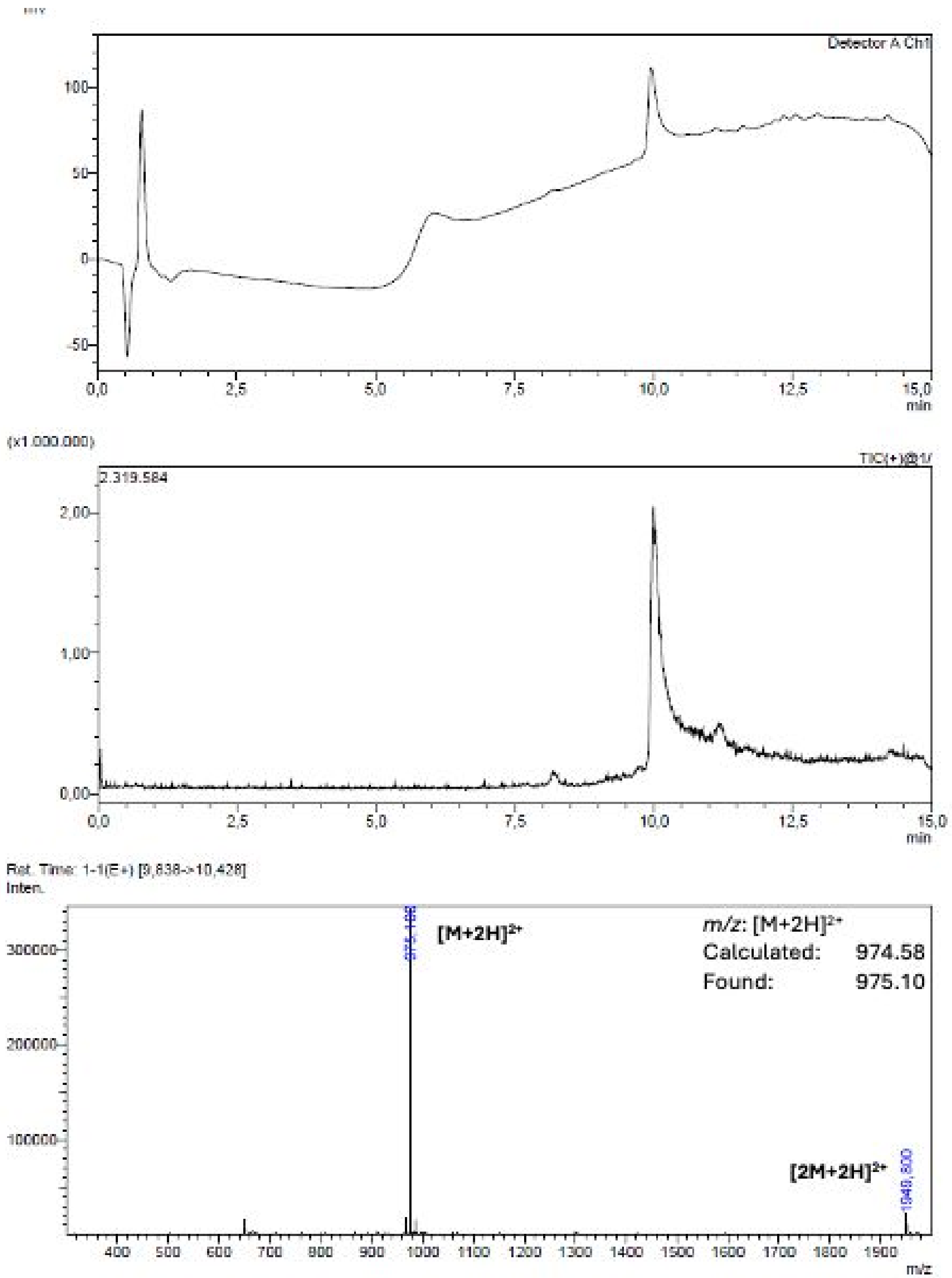

**Figure S14: LC-MS Analysis MDM2_B12**

**MDM2_B12:**
Ac-K(Biotin)-βA-SQFRKLWEEVMSGKALE -CONH$_2$

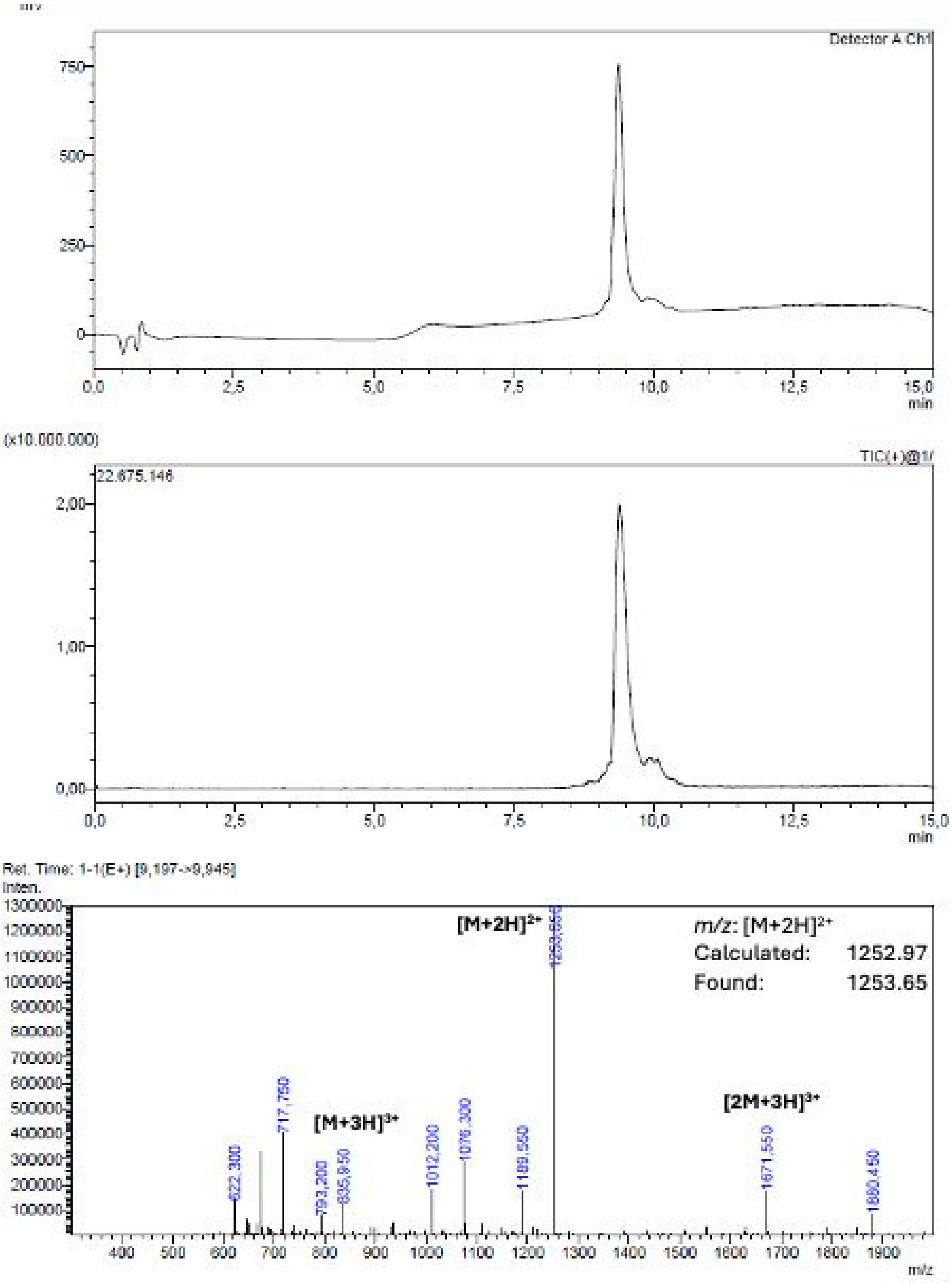

**Figure S15: LC-MS Analysis MDM2_B13**

**MDM2_B13:**
Ac-K(Biotin)-βA-SQFRELWEEVMSGKALE-$CONH_2$

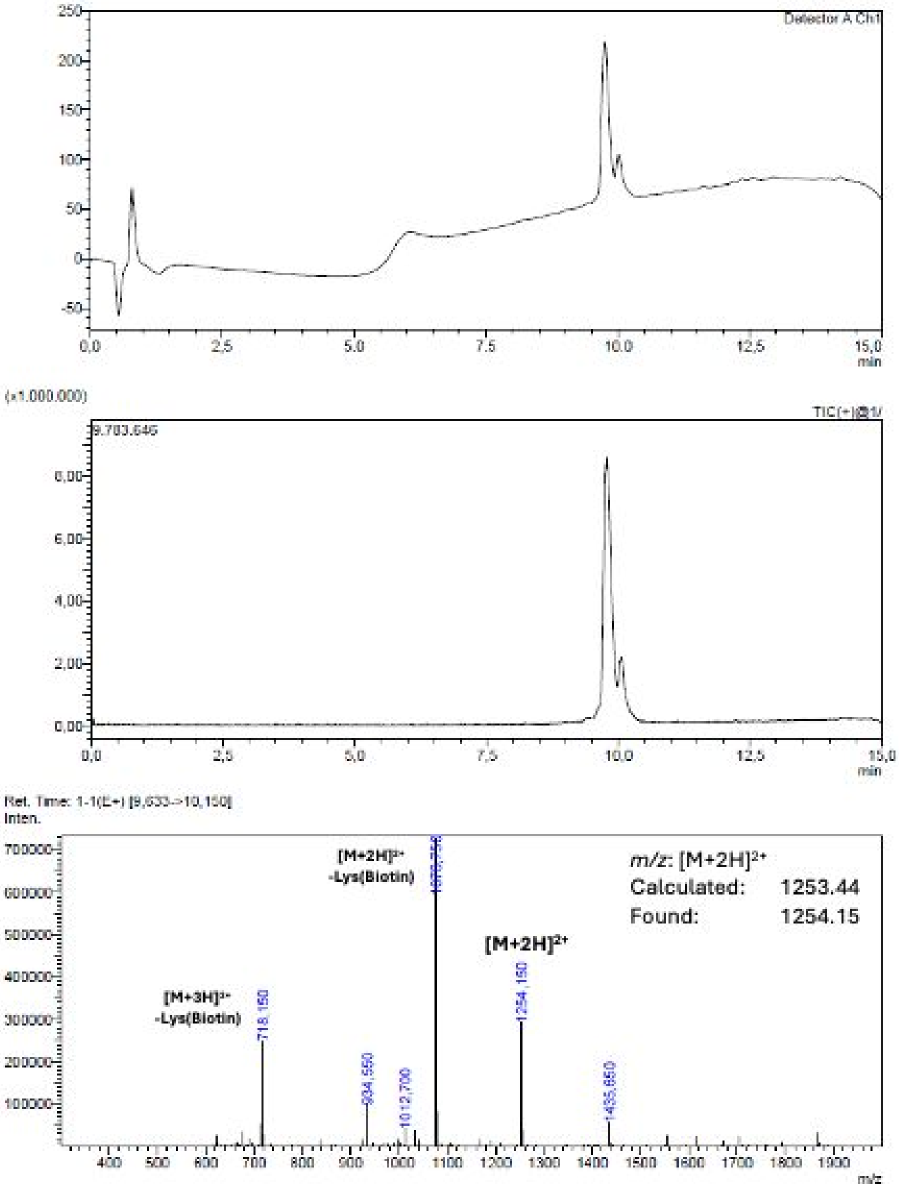

**Figure S16: LC-MS Analysis MDM2_B14**

**MDM2_B14:**

Ac-K(Biotin)-βA-TGFQKEWEKVMNS-$CONH_2$

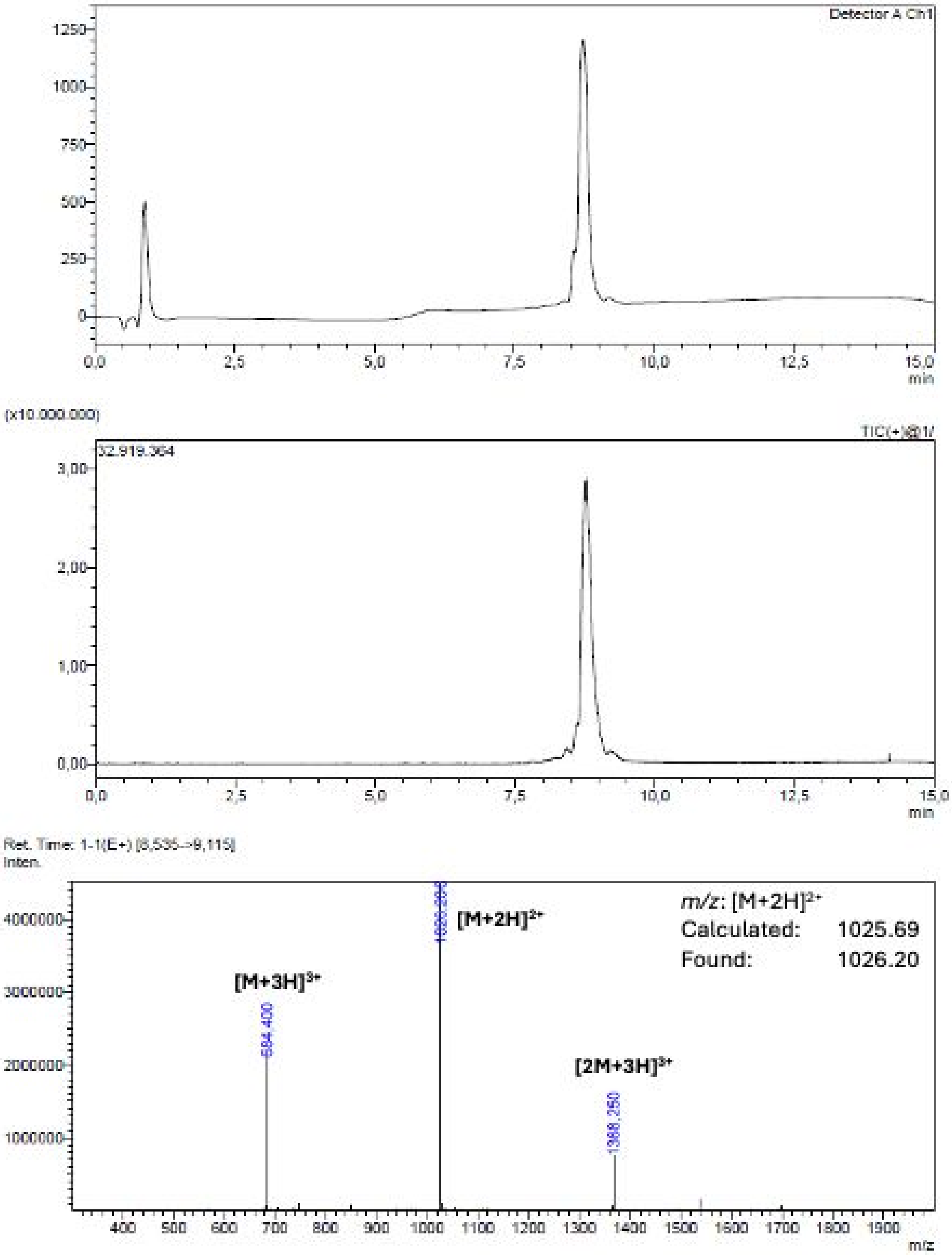

**Figure S17: LC-MS Analysis MDM2_B15**

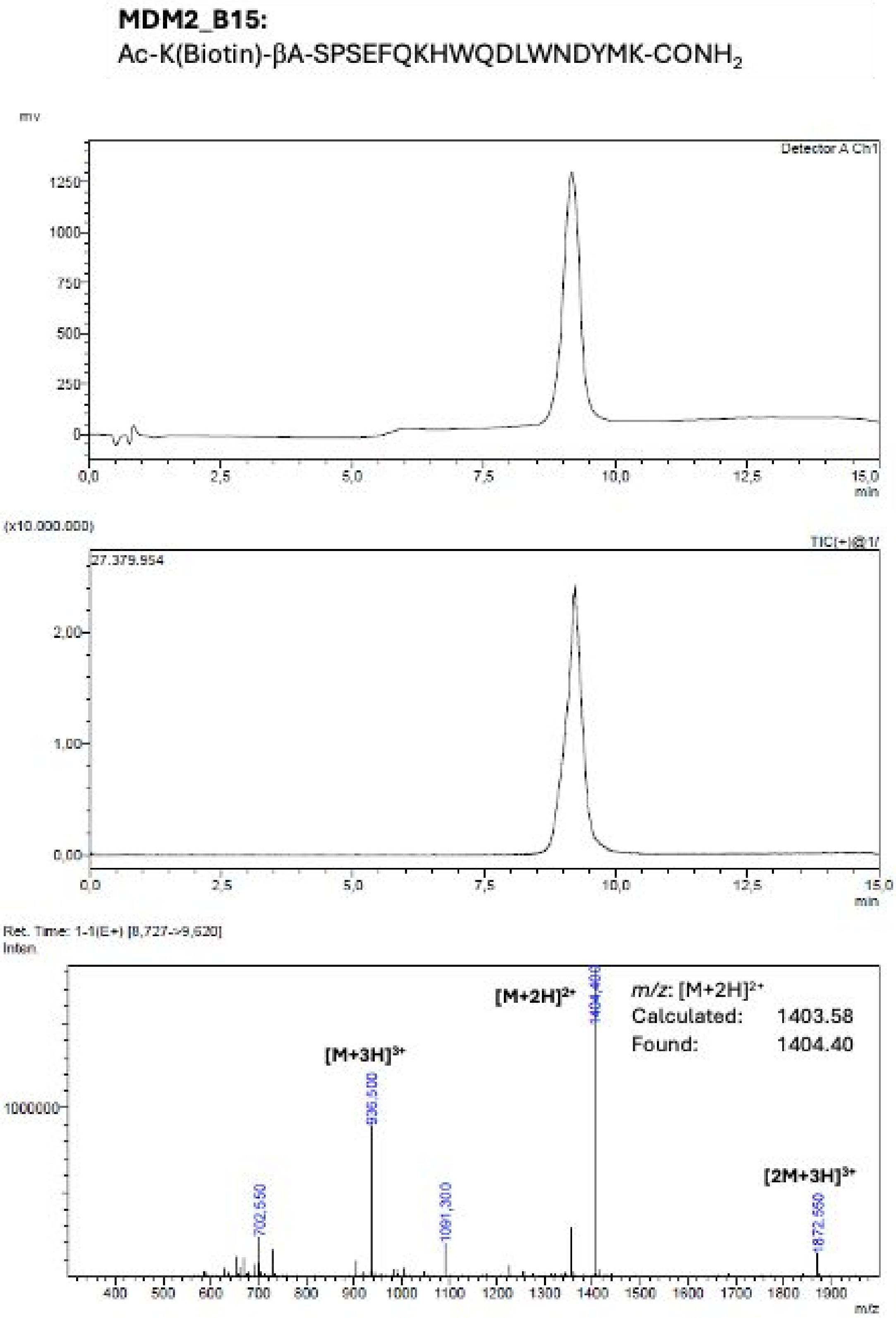

**Figure S18: LC-MS Analysis MDM2_B17**

**MDM2_B17:**
Ac-K(Biotin)-βA-SPSEFQKHWQDLWDDYMK-$CONH_2$

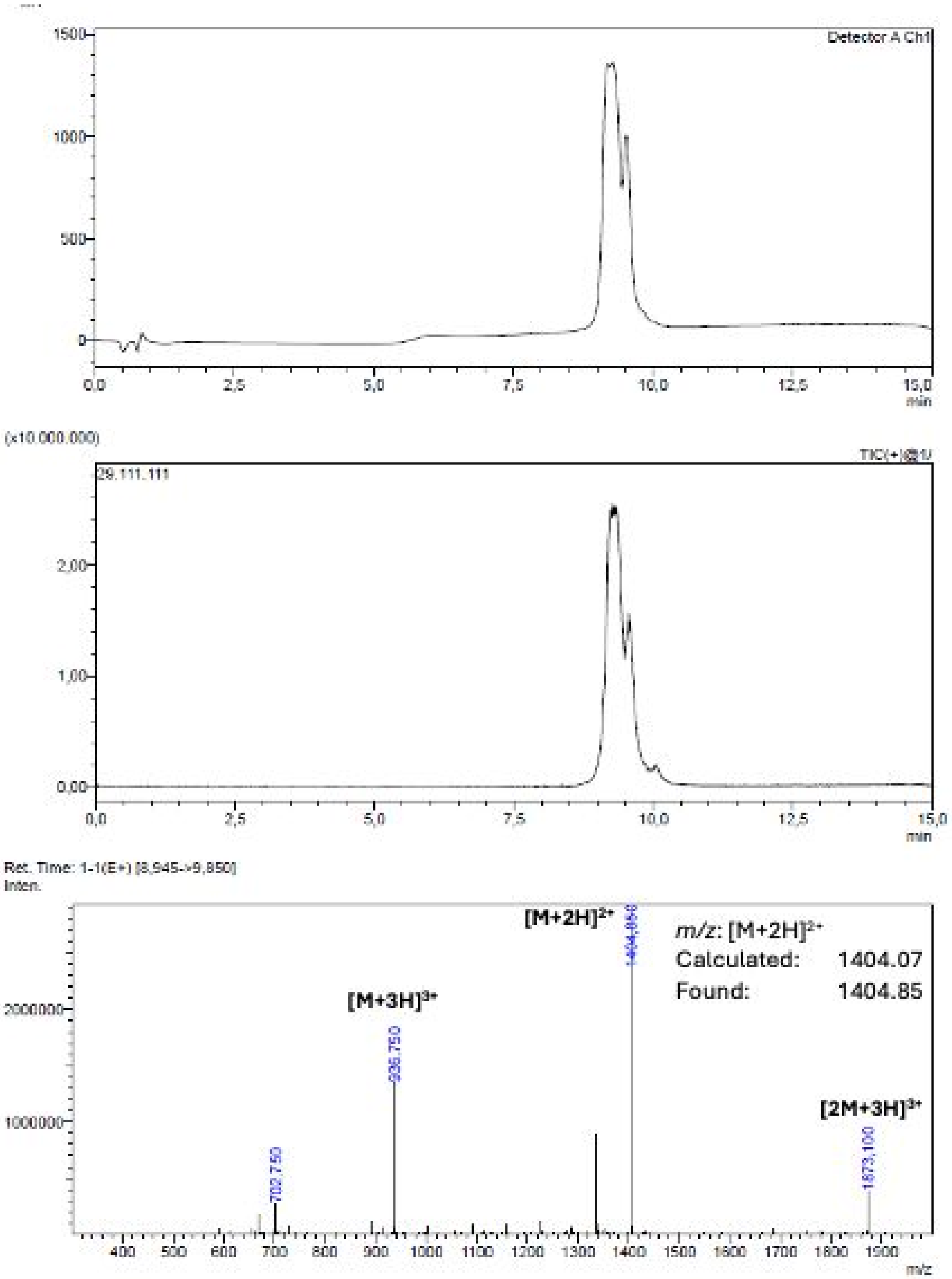

**Figure S19: LC-MS Analysis MDM2_B18**

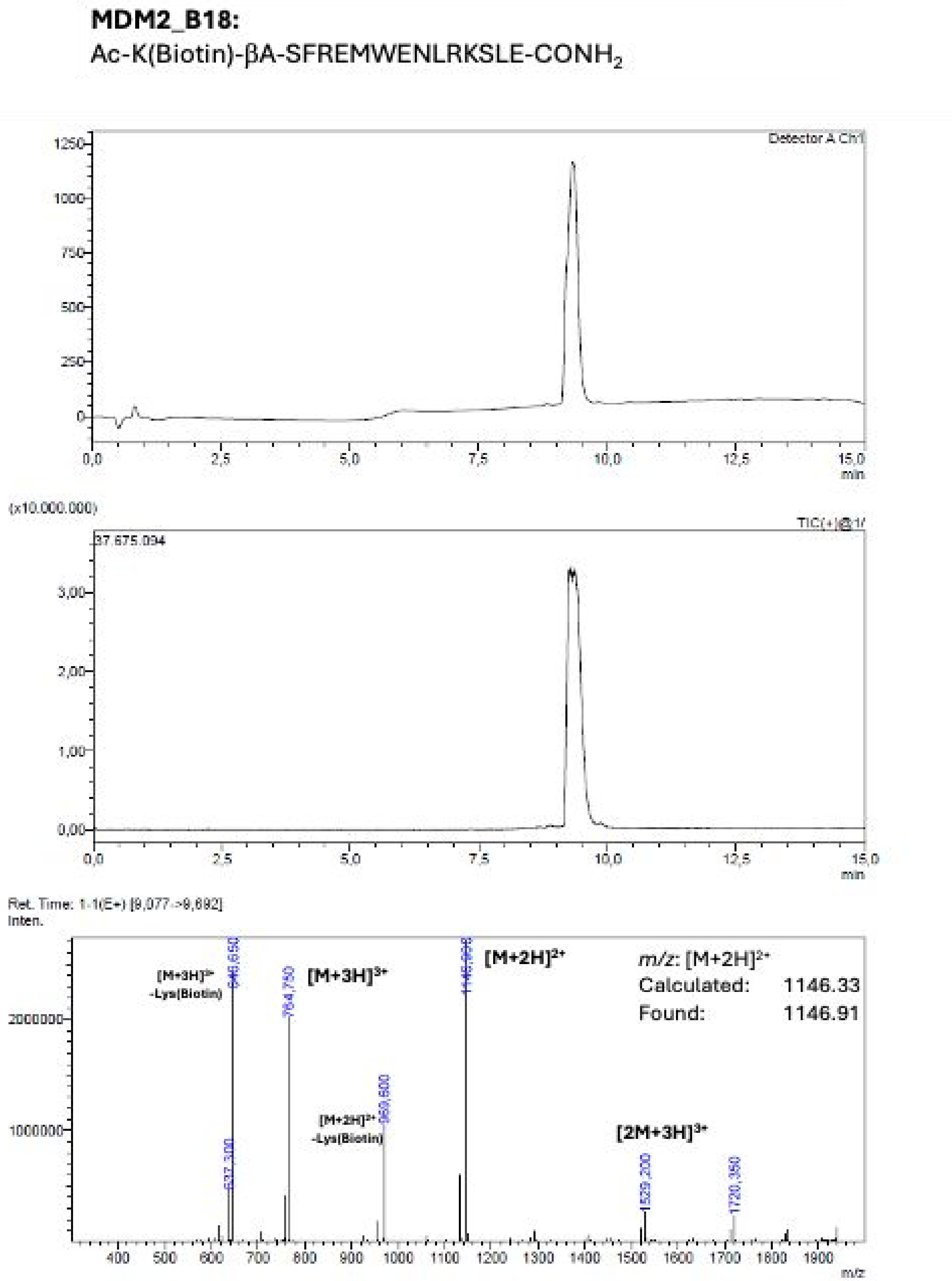

**Figure S20: LC-MS Analysis MDM2_B19**

**MDM2_B19:**
Ac-K(Biotin)-βA-SFRDMWENLRKSLE-$CONH_2$

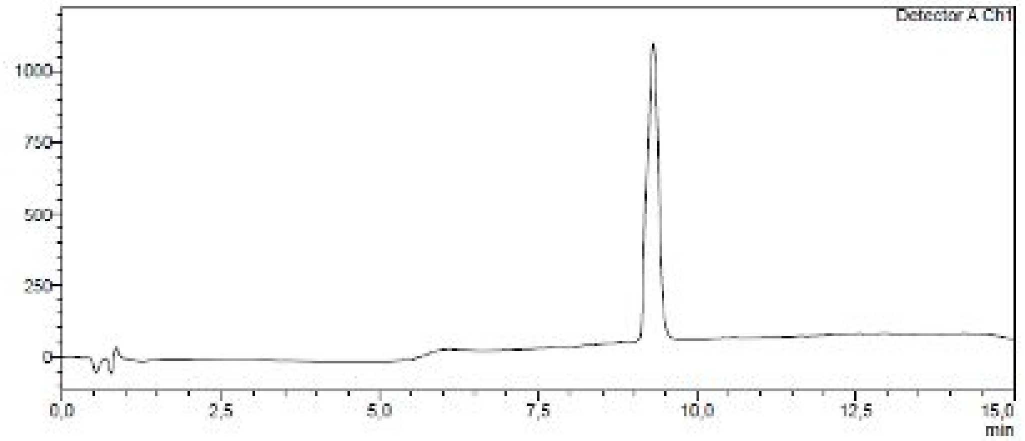


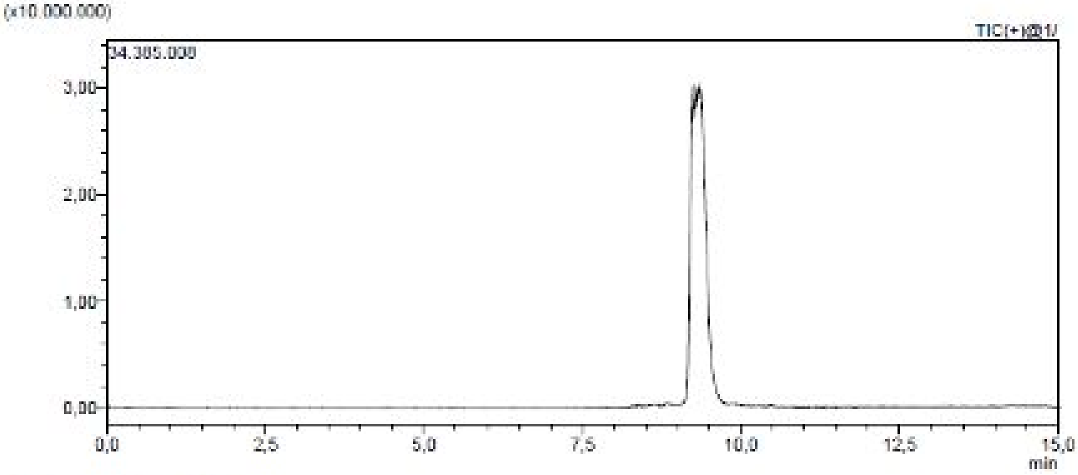


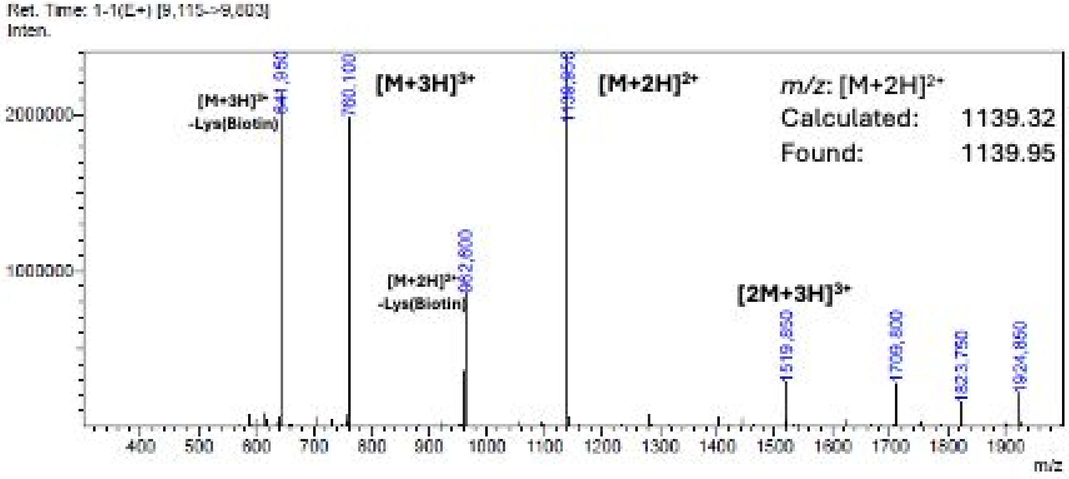

**Figure S21: LC-MS Analysis MDM2_B20**

**MDM2_B20:**

Ac-K(Biotin)-βA-GWEGFMKQWKEFSENLEKYM-$CONH_2$

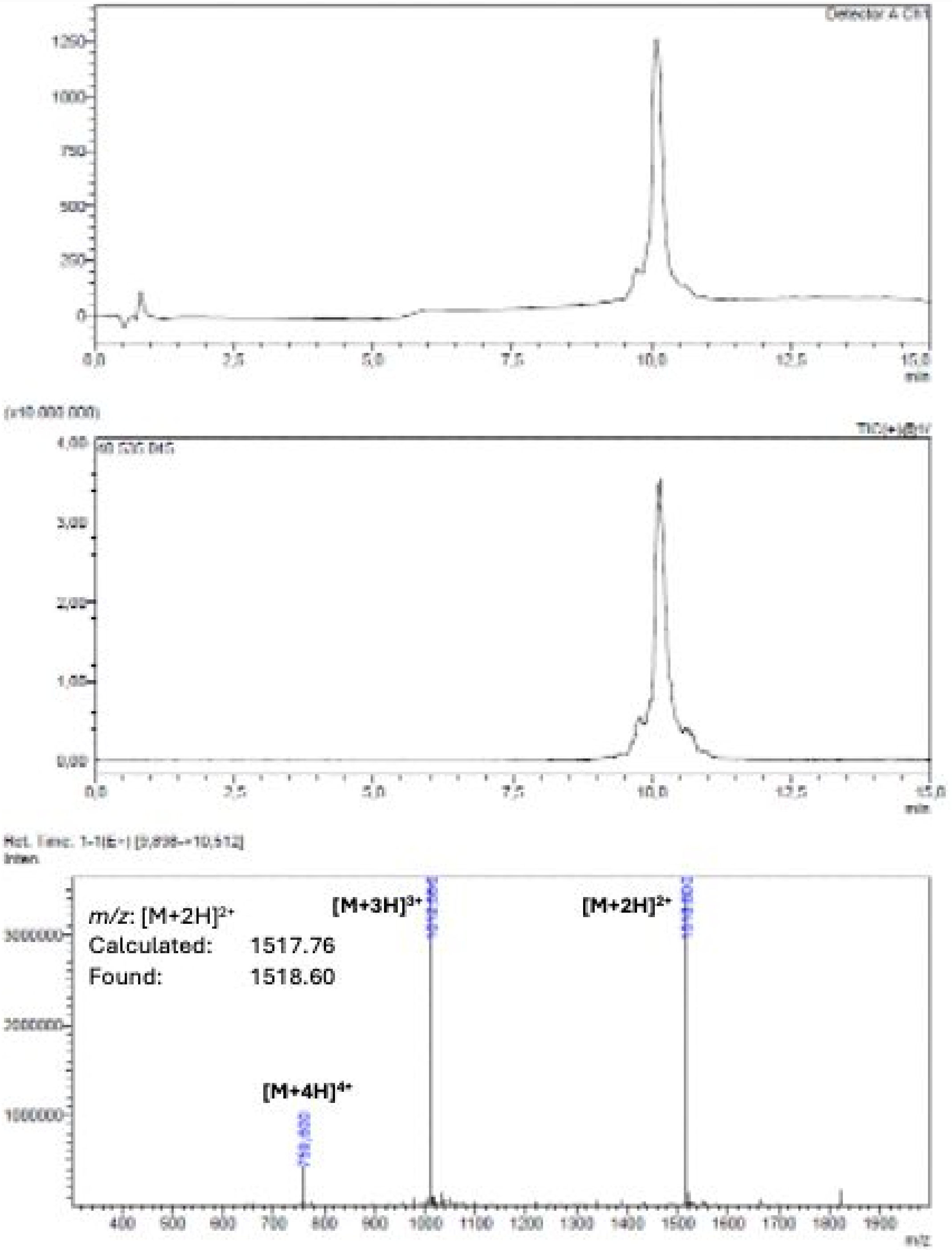

**Figure S22: LC-MS Analysis WDR5_MYC_B1**

**WDR5_Myc_B1:**
Ac-K(Biotin)-βA-DDEDFEQFMKDLDEFLK-$CONH_2$

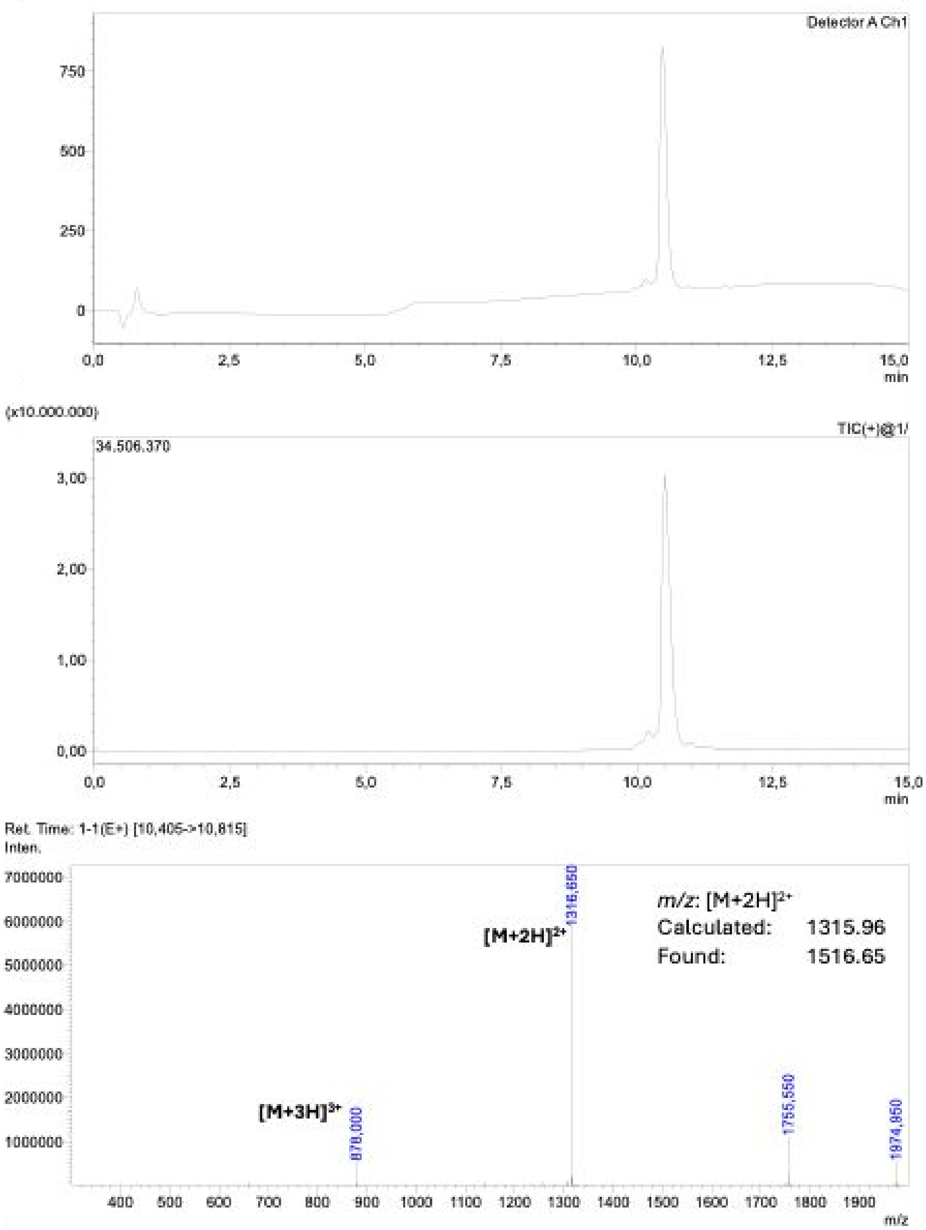

**Figure S23: LC-MS Analysis WDR5_MYC_B3**

**WDR5_Myc_B3:**
Ac-K(Biotin)-βA-DTDKVLEQIEKEQ-$CONH_2$

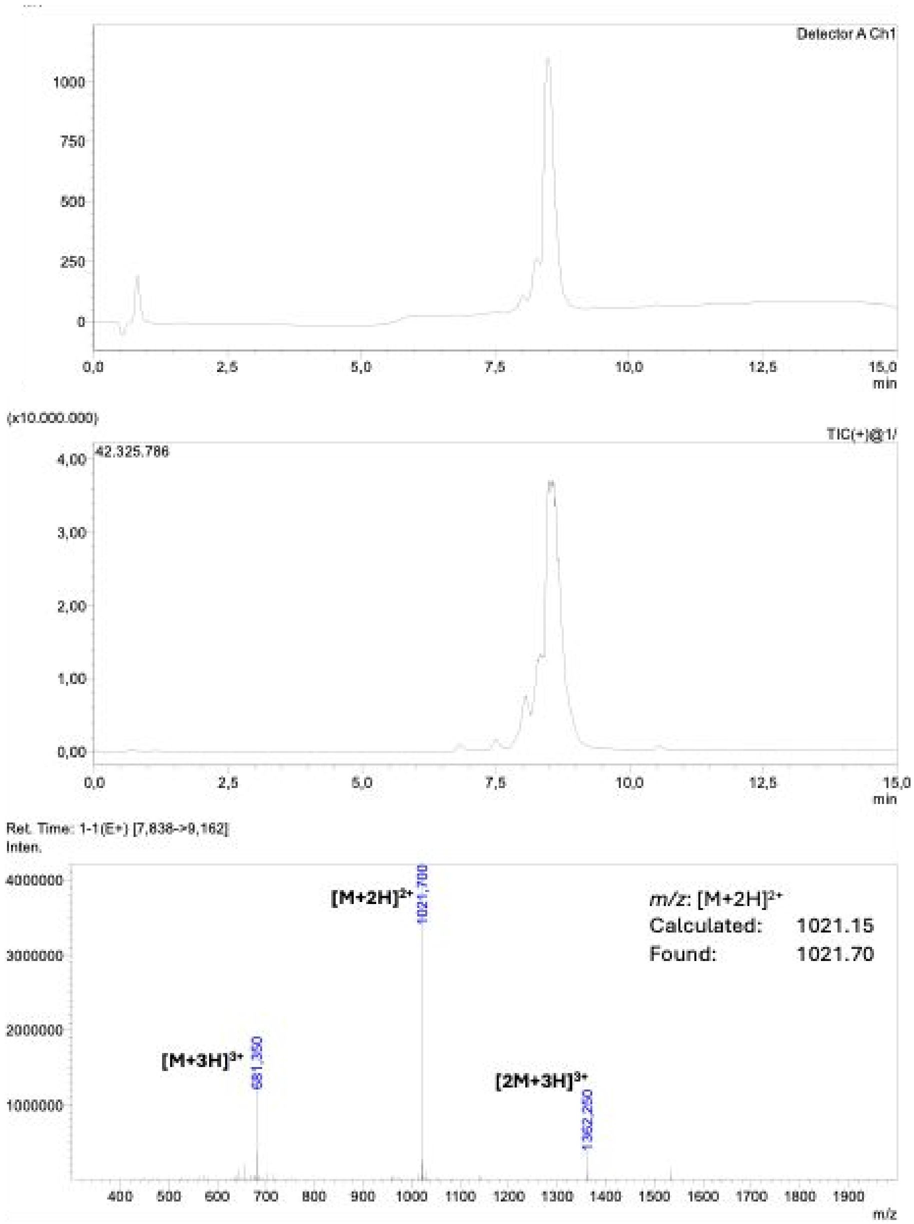

**Figure S24: LC-MS Analysis WDR5_MYC_B5**

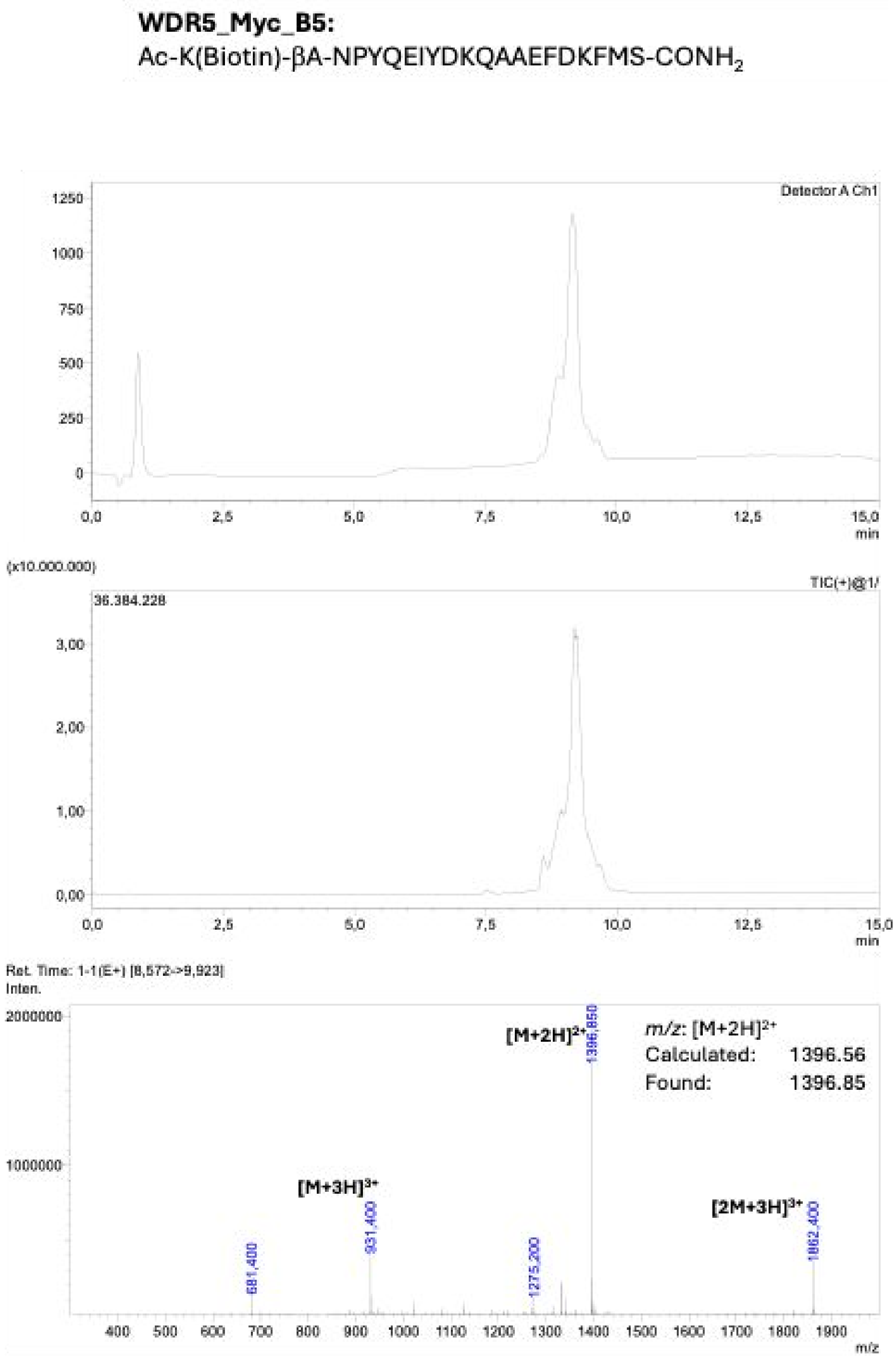

**Figure S25: LC-MS Analysis WDR5_MYC_B6**

**WDR5_Myc_B6:**

Ac-K(Biotin)-βA-SAEEDEKAMQELMDYLNSM-$CONH_2$

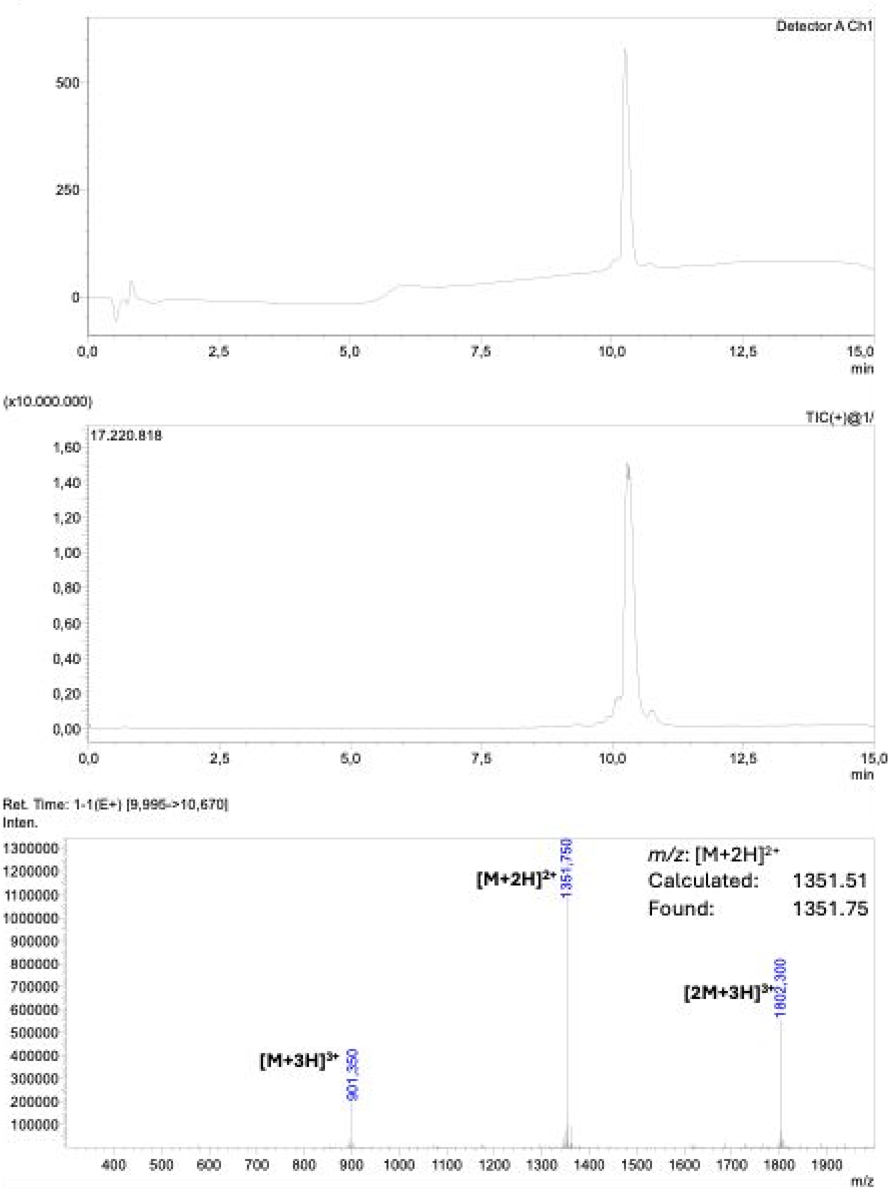

**Figure S26: LC-MS Analysis WDR5_MYC_B7**

**WDR5_Myc_B7:**
Ac-K(Biotin)-βA-DPEVDELIKFMDENADAWR-$CONH_2$

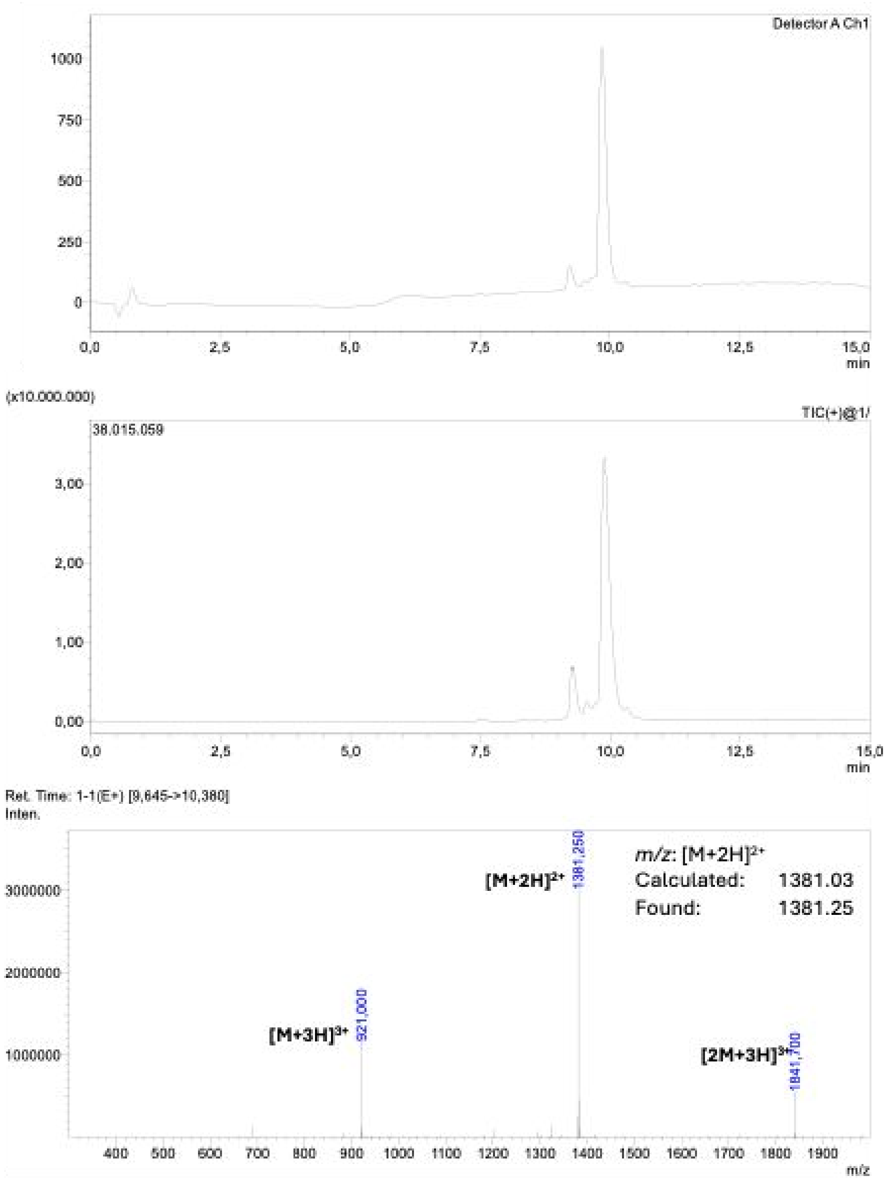

**Figure S27: LC-MS Analysis WDR5_MYC_B8**

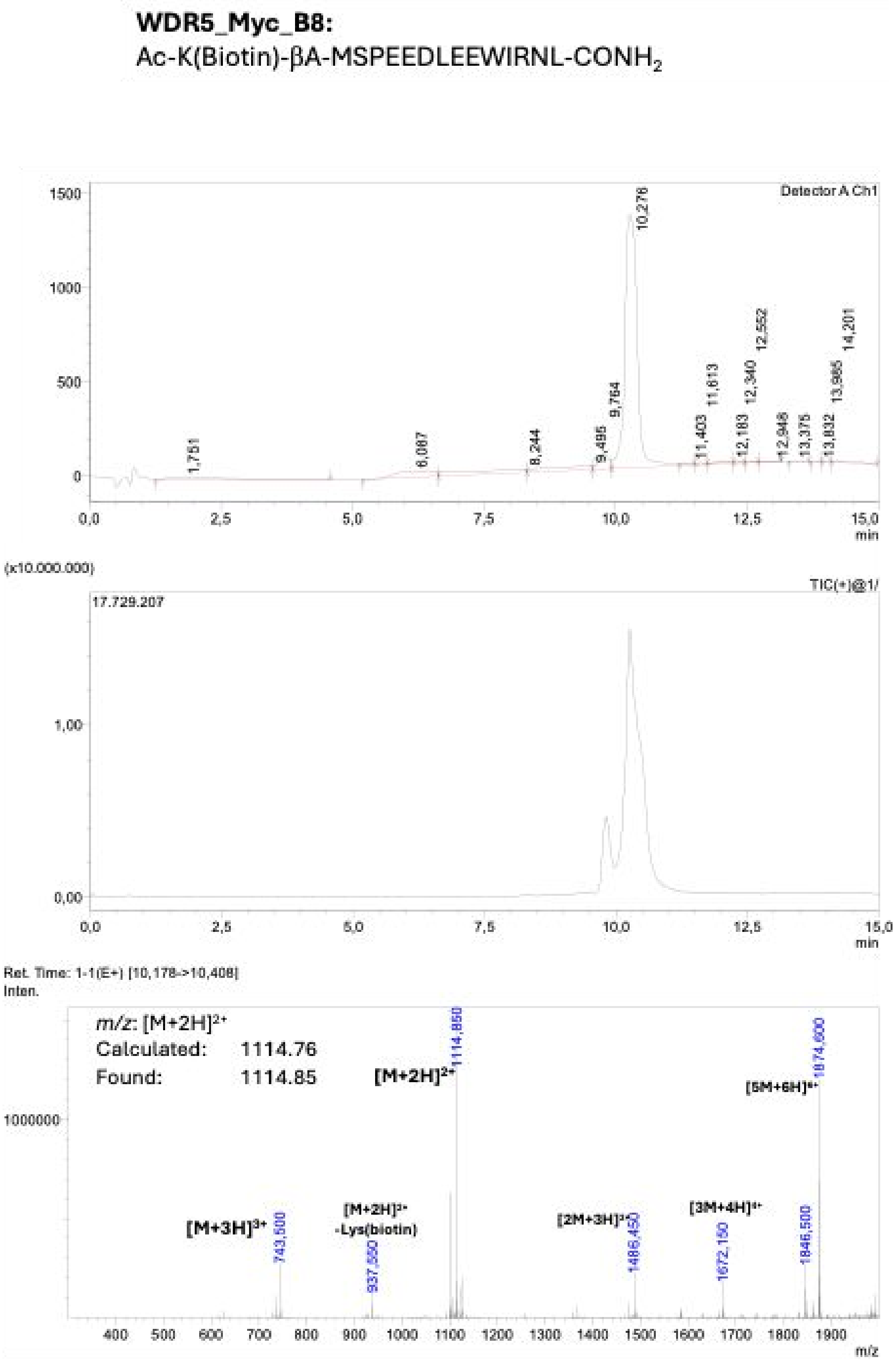

**Figure S28: LC-MS Analysis WDR5_MYC_B10**

**WDR5_Myc_B10:**

Ac-K(Biotin)-βA-SPDKDVQELIDYLNS-$CONH_2$

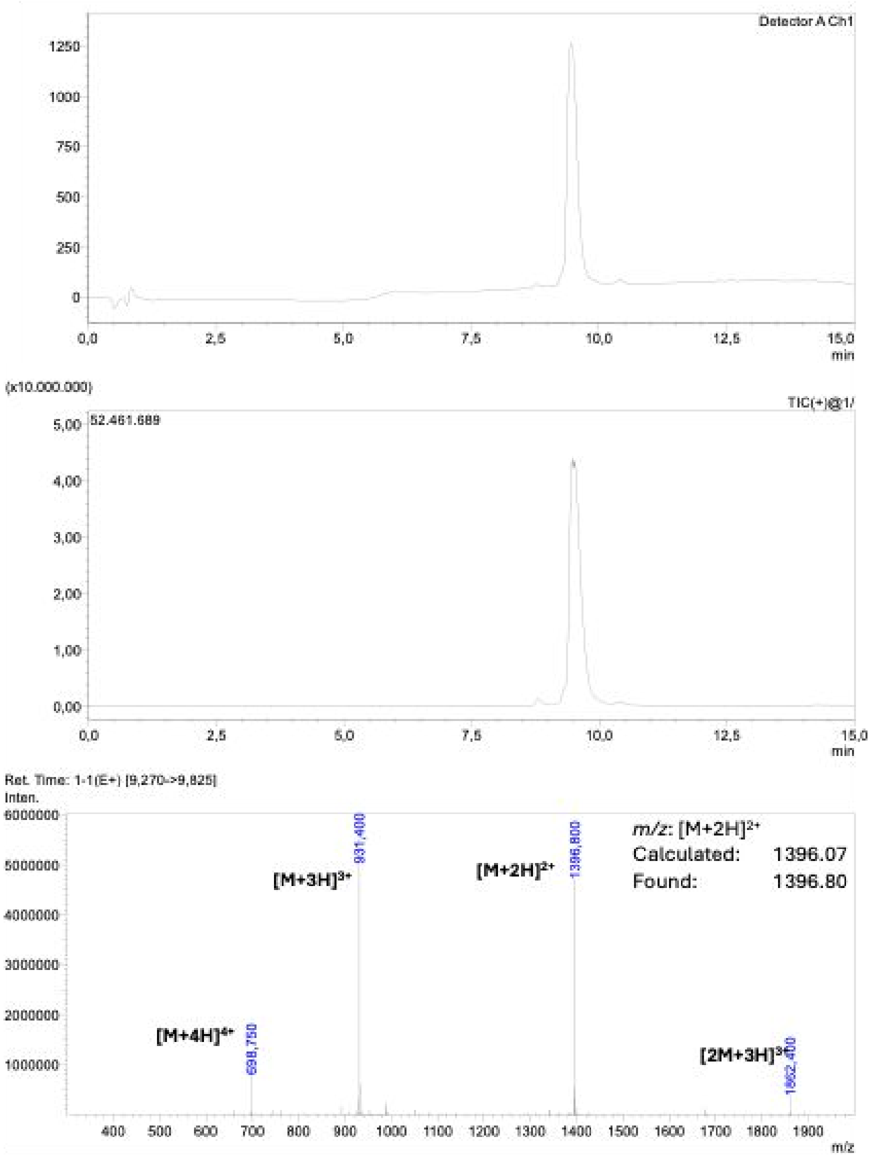

**Figure S29: LC-MS Analysis WDR5_MYC_B11**

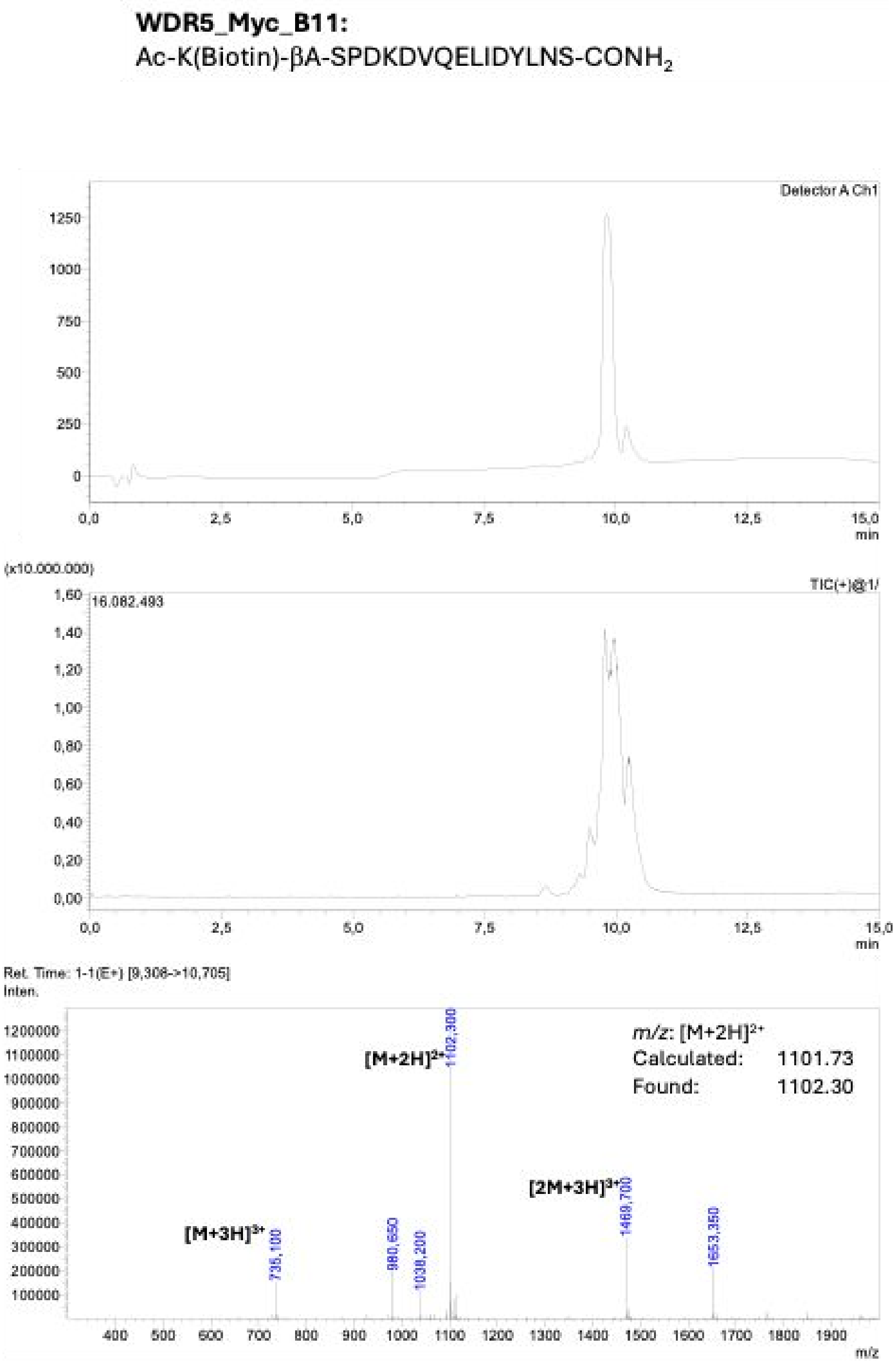

**Figure S30: LC-MS Analysis WDR5_MYC_B12**

**WDR5_Myc_B12:**

Ac-K(Biotin)-βA-DTDEVLEQIEKEQ-$CONH_2$

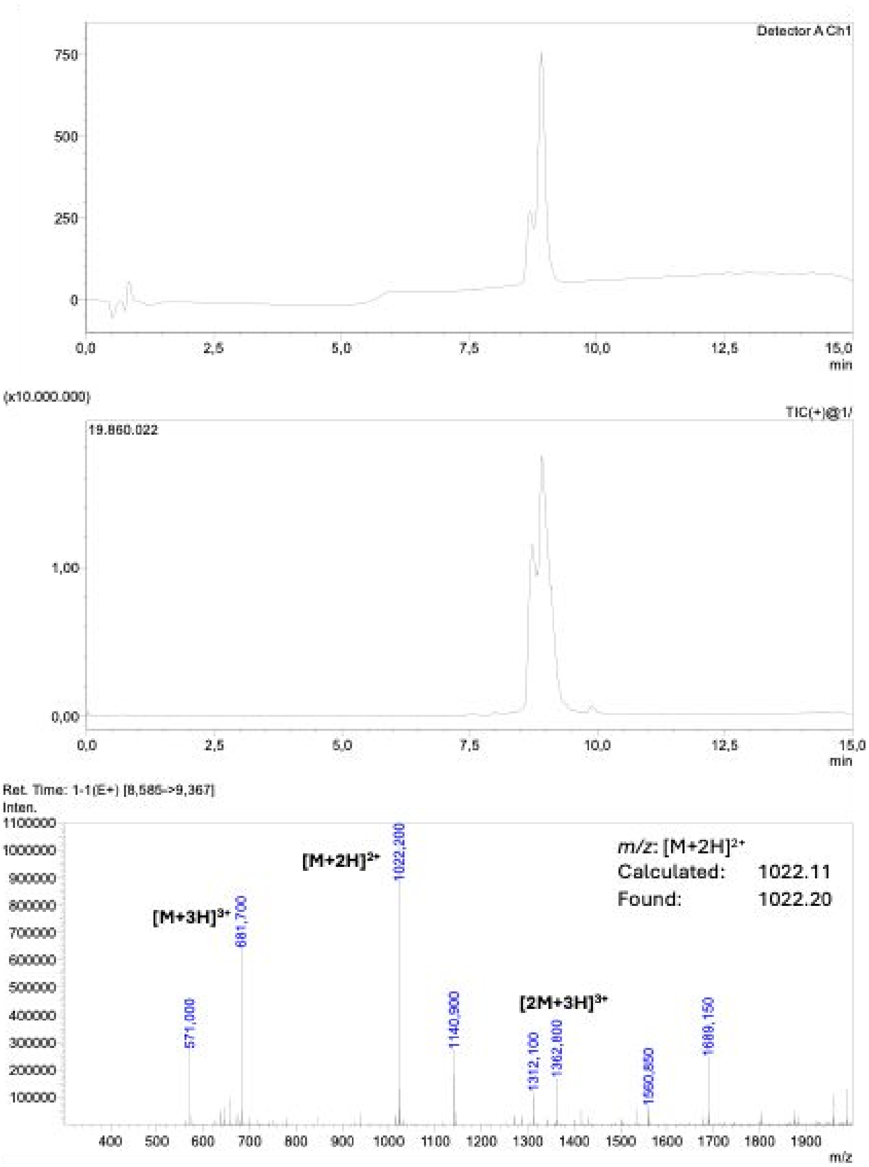

## Figure S31: LC-MS Analysis WDR5_WIN_B2

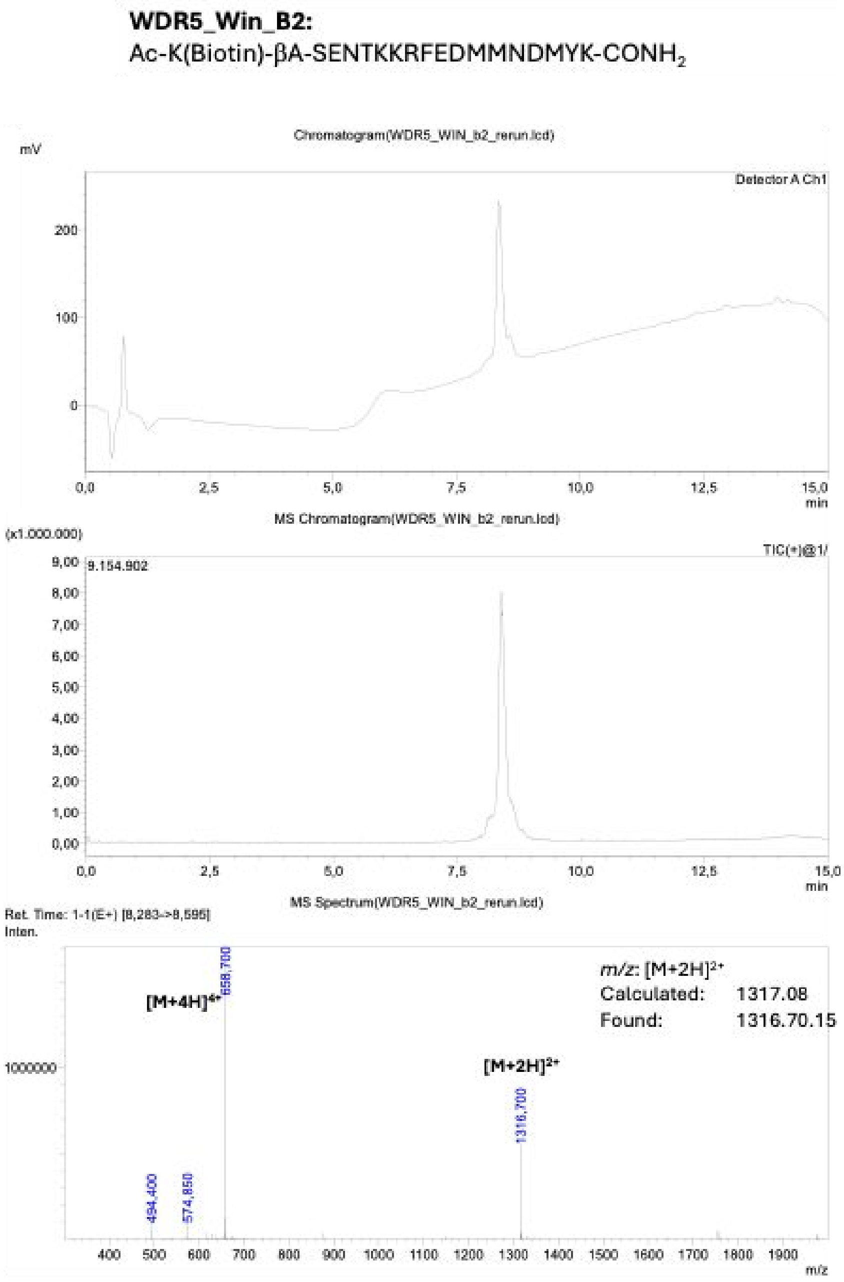

**Figure S32: LC-MS Analysis WDR5_WIN_B9**

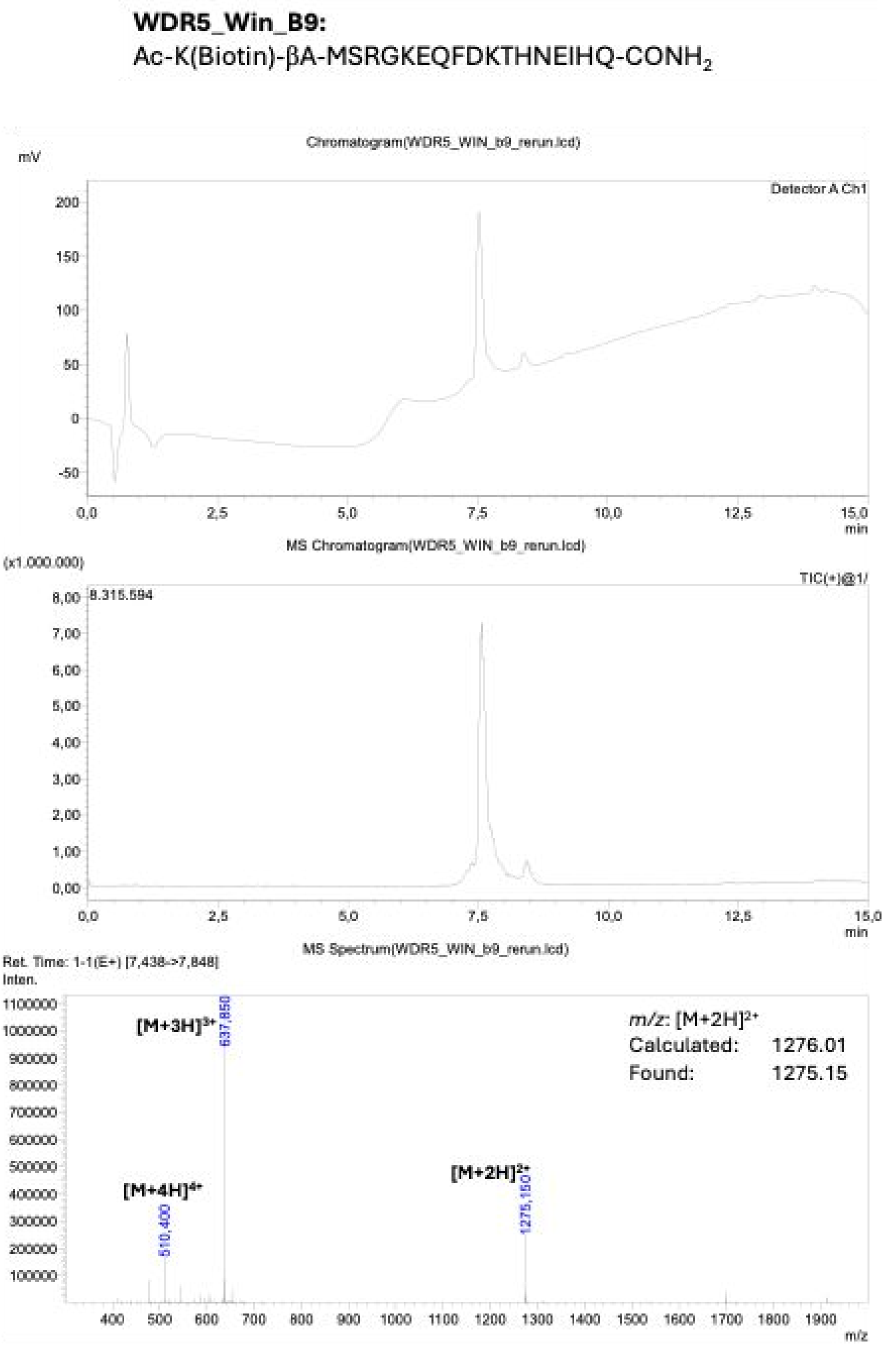

**Figure S33: LC-MS Analysis WDR5_WIN_B10**

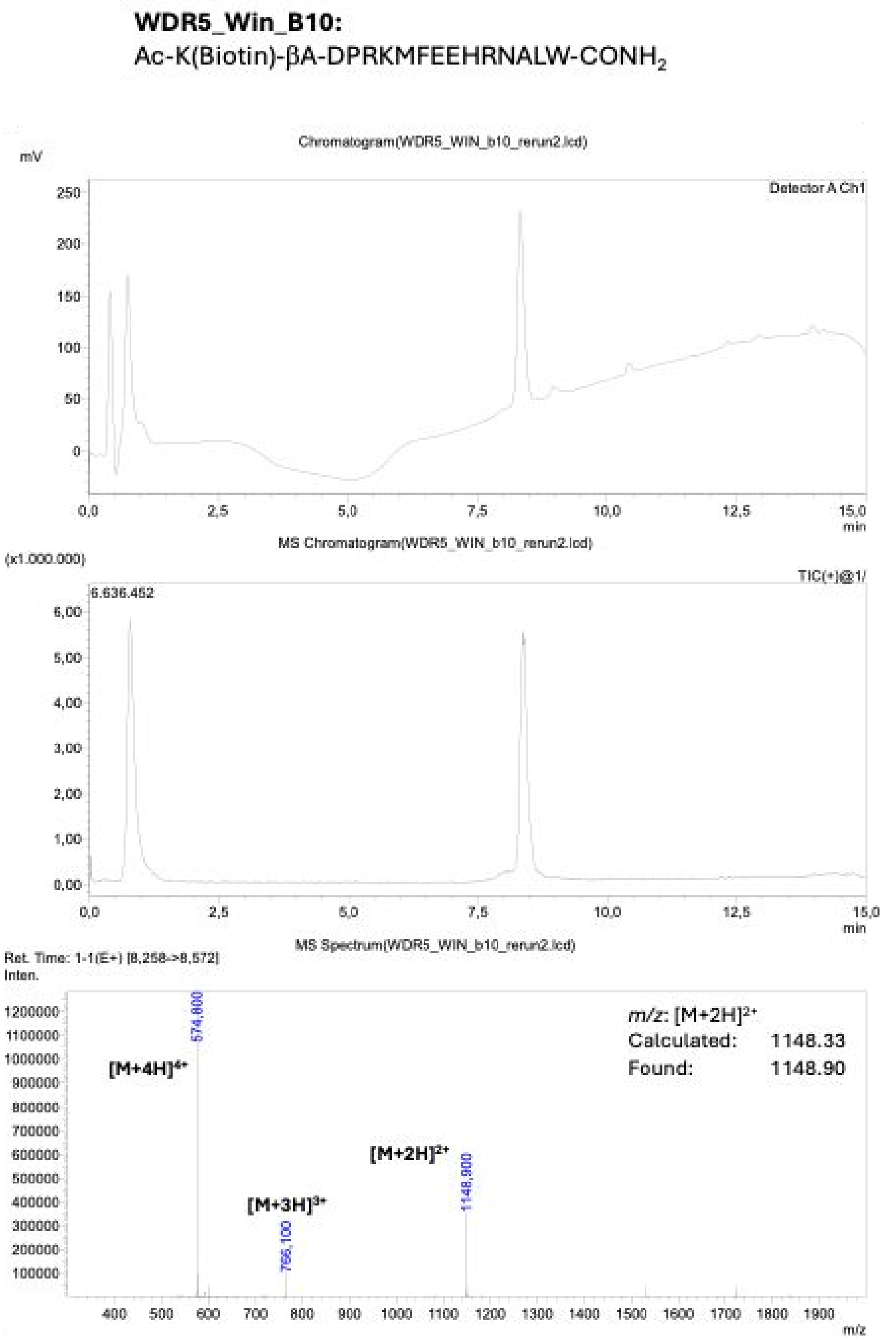

**Figure S34: LC-MS Analysis WDR5_WIN_B11**

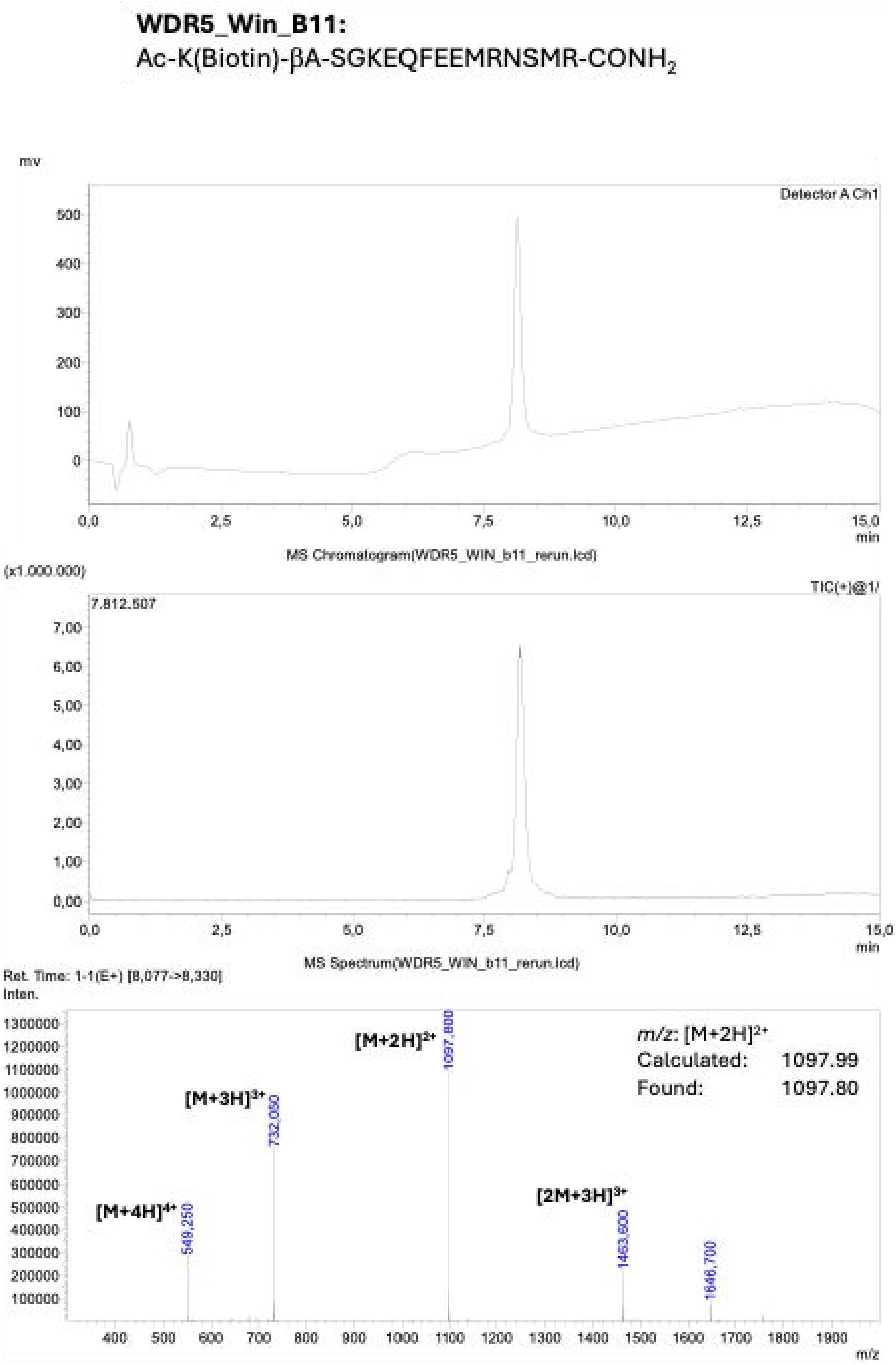

**Figure S35: LC-MS Analysis Myc_peptide_Motif.** Peptide used for WDR5-Myc competition assay.

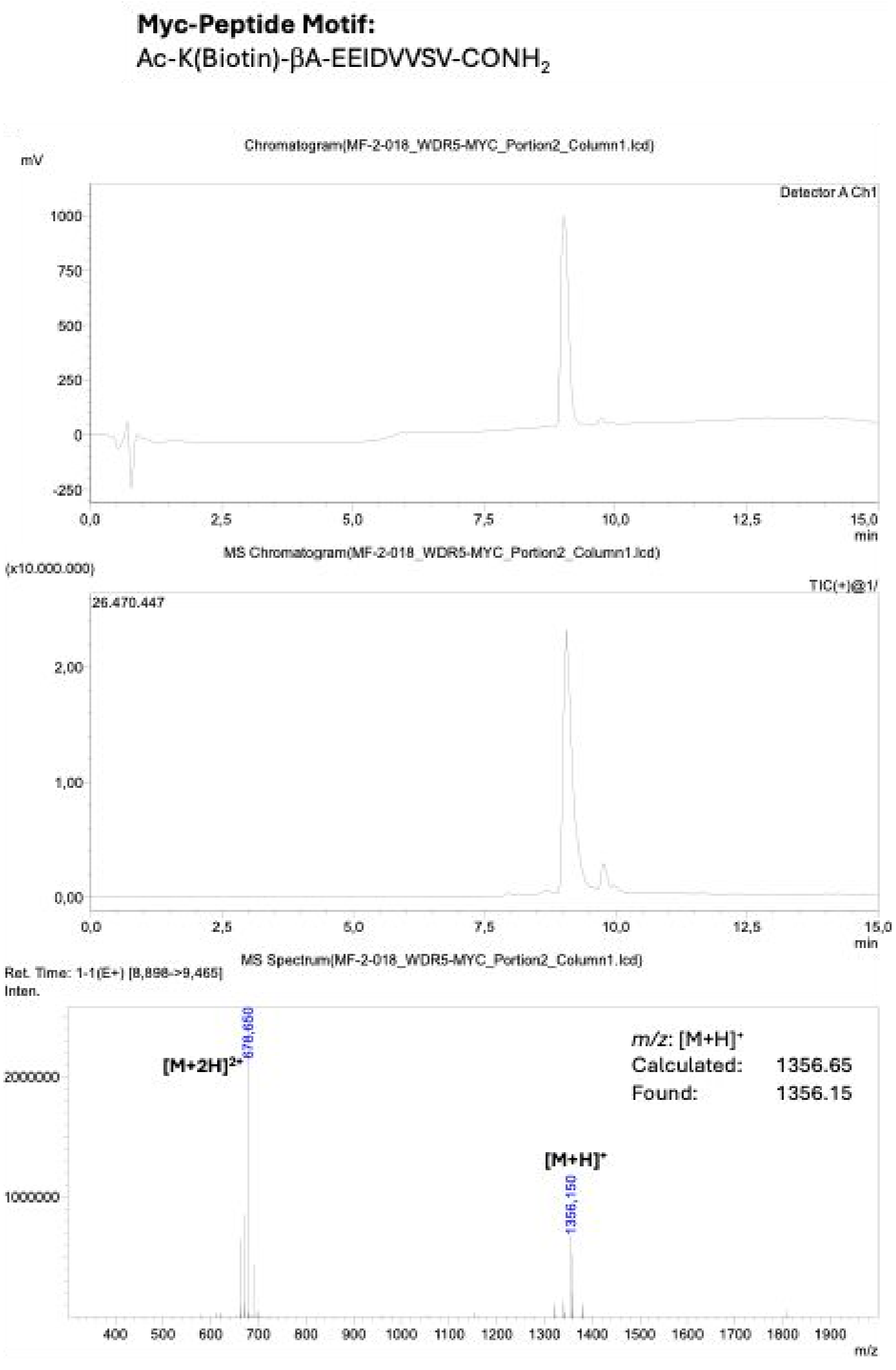

**Figure S36: LC-MS Analysis p53_peptide_Motif.** Peptide used for MDM2-p53 competition assay.

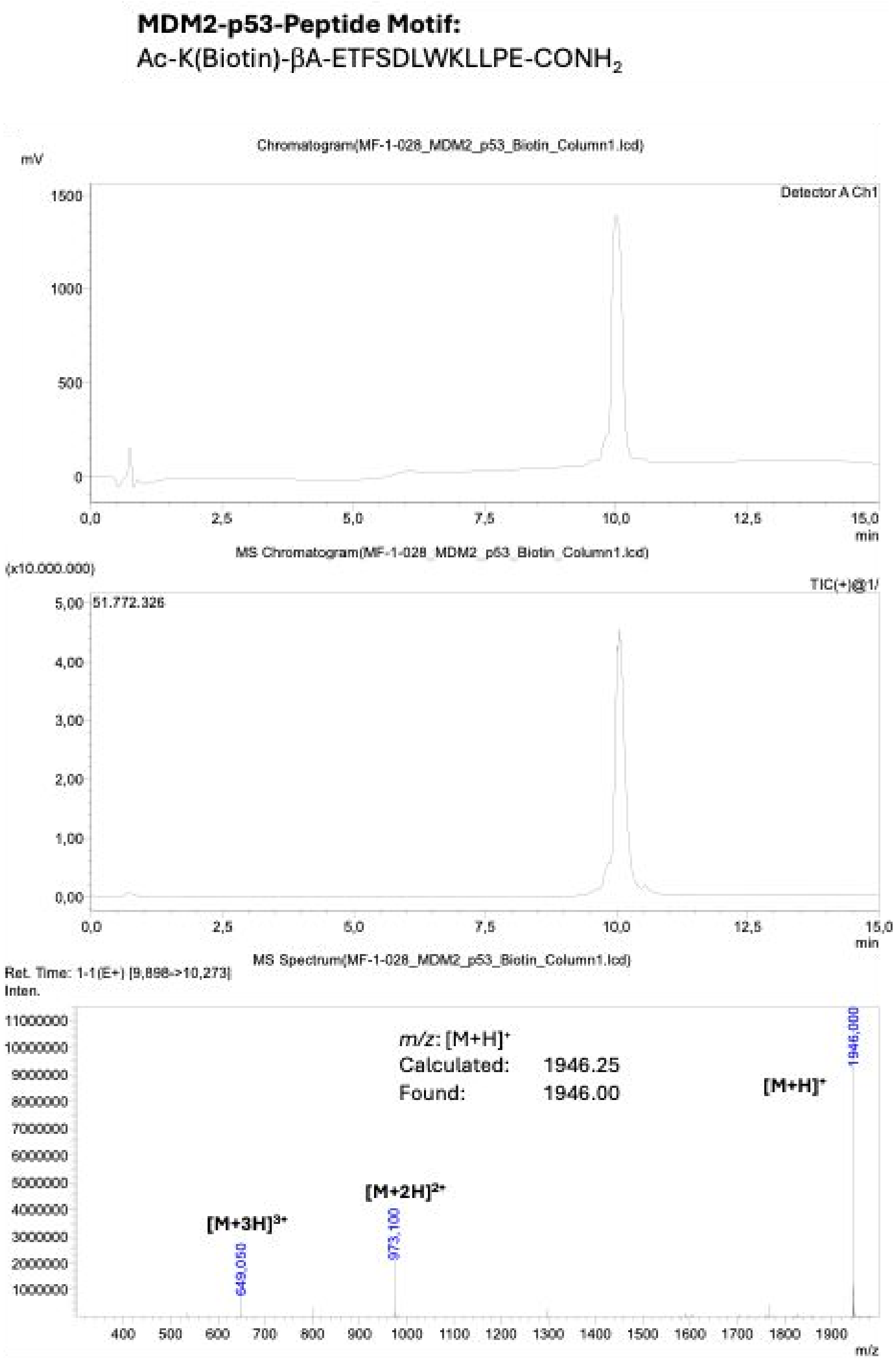

**Figure S37: LC-MS Analysis MDM2_B1_compeition peptide.** Peptide used for MDM2 competition assay.

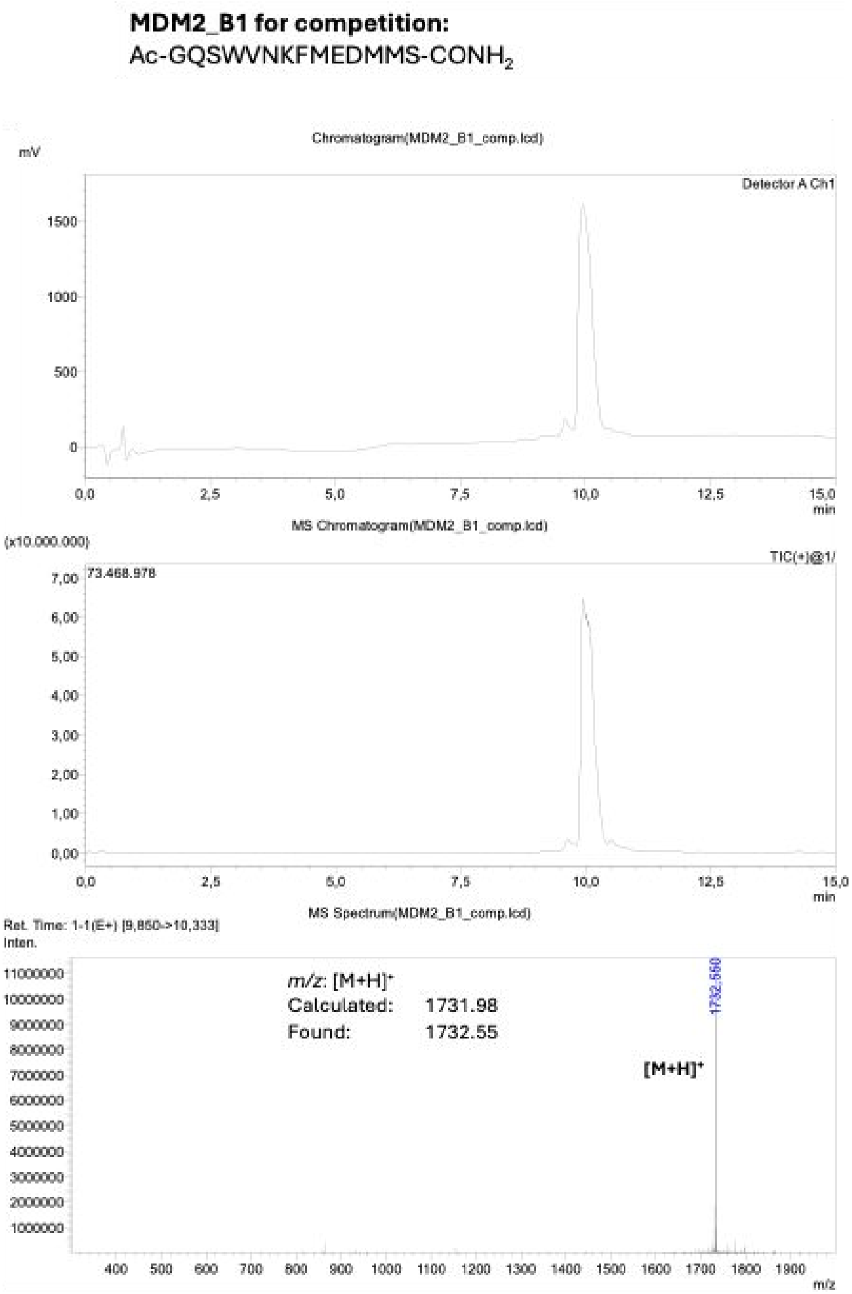

**Figure S38: LC-MS Analysis MDM2_B1_compeition peptide.** Peptide used for MDM2 competition assay.

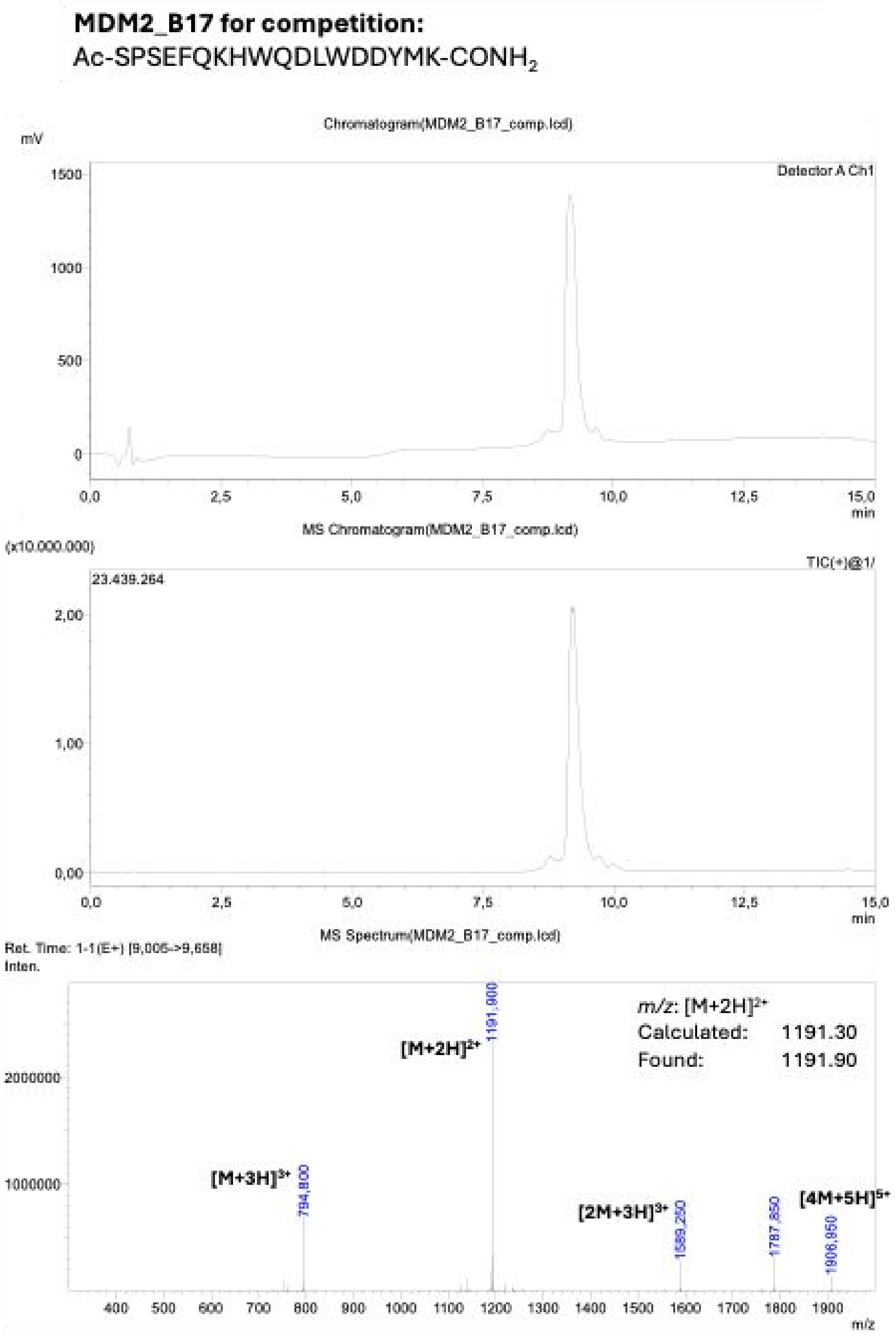

**Figure S39: LC-MS Analysis WDR5_Myc_B1_compeition peptide.** Peptide used for WDR5 competition assay.

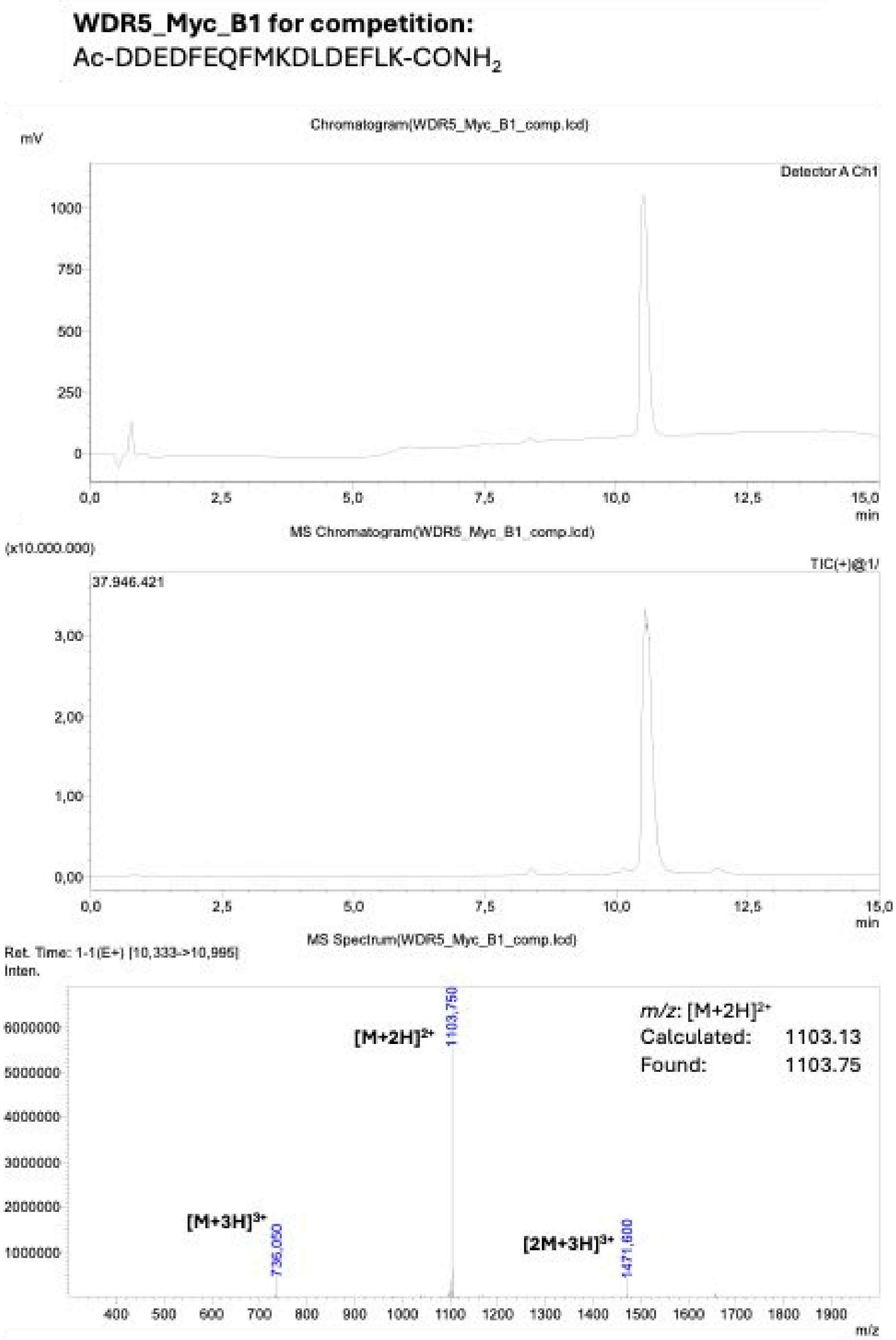

**Figure S40: LC-MS Analysis WDR5_Myc_B1_stapling_biotin.**

**WDR5_Myc_B1_biotin_stapled:**

Ac-K(Biotin)-βA-DDEDF**C**QFM**C**DLDEFLK-$CONH_2$

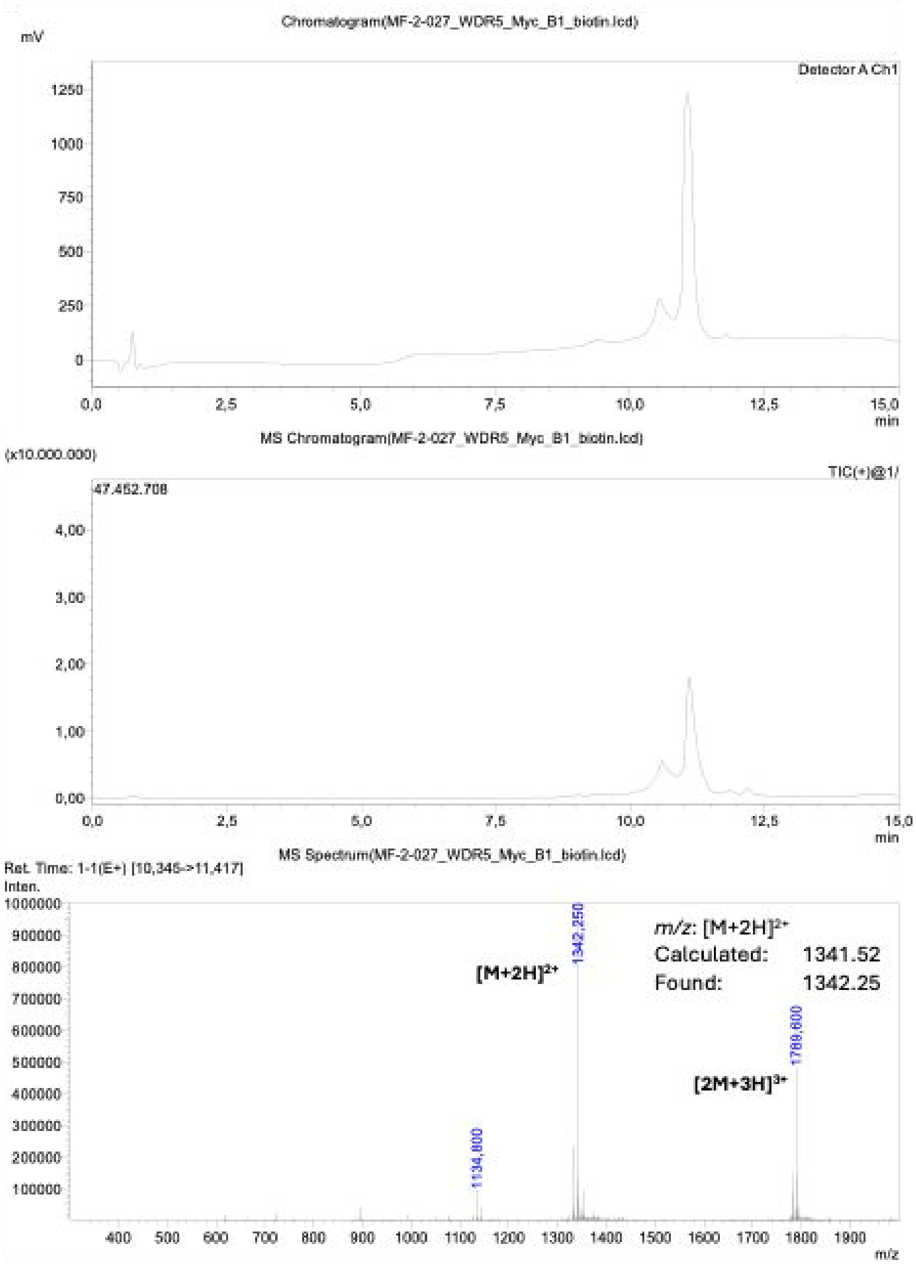

**Figure S41: LC-MS Analysis WDR5_Myc_B1_stapling competition assay.**

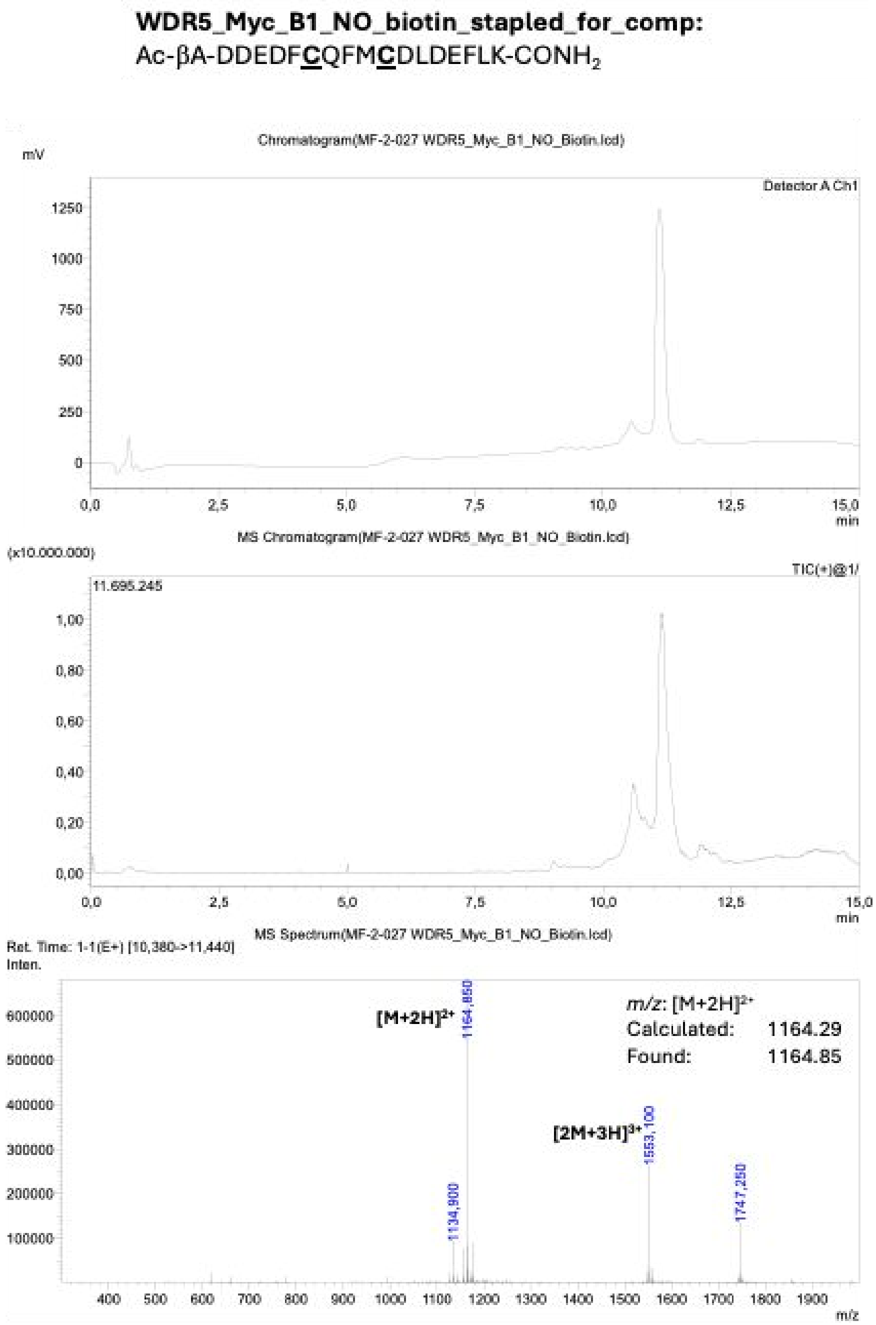

**Figure S42: LC-MS Analysis PDL1_B1**

**PDL1_B1:**

Ac-K(Biotin)-βA-PSGMEVFQRLVEEQRA-$CONH_2$

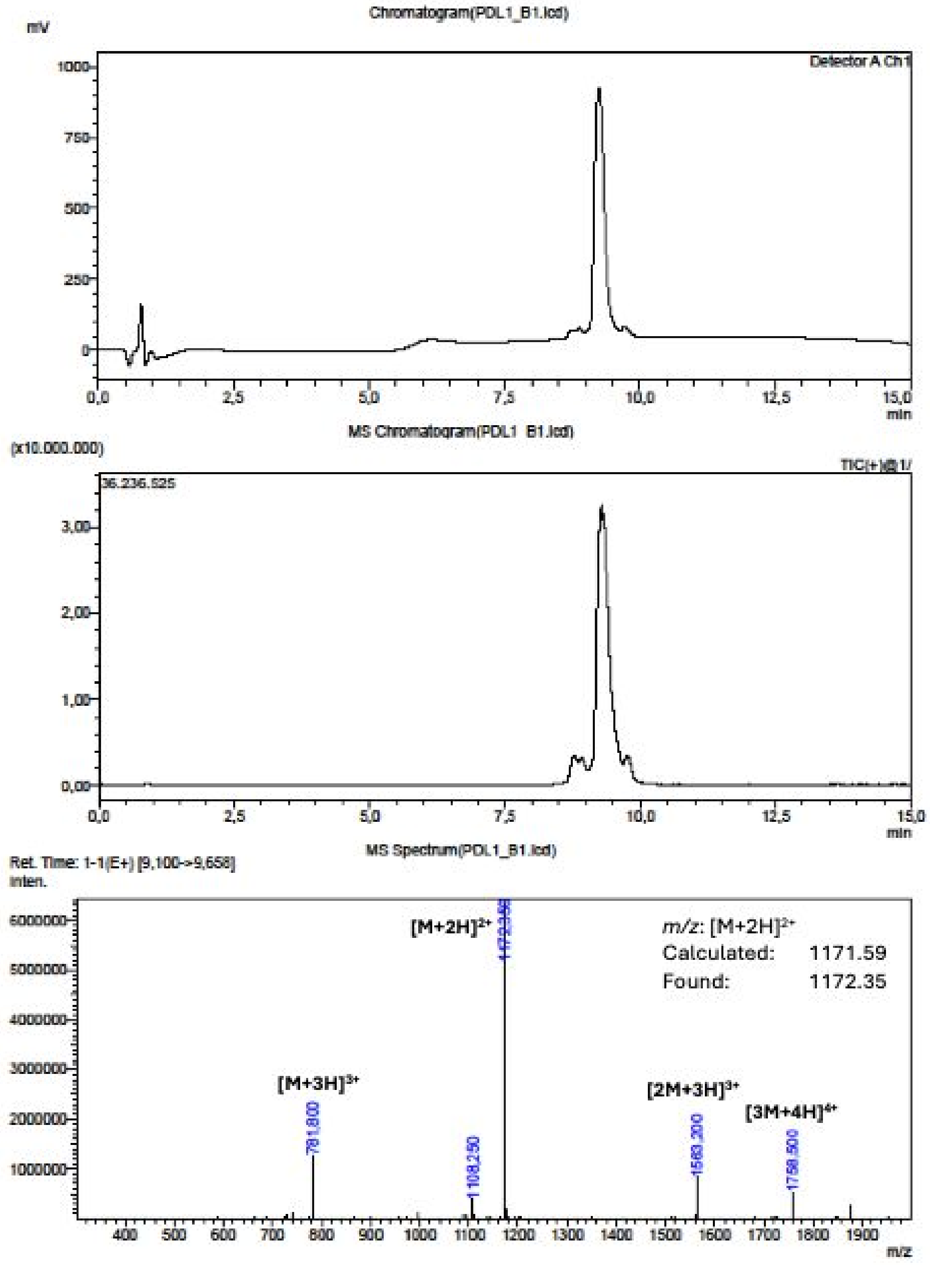

**Figure S43: LC-MS Analysis PDL1_B2**

**PDL1_B2:**

Ac-K(Biotin)-βA-SVKDLMEQLIAS-$CONH_2$

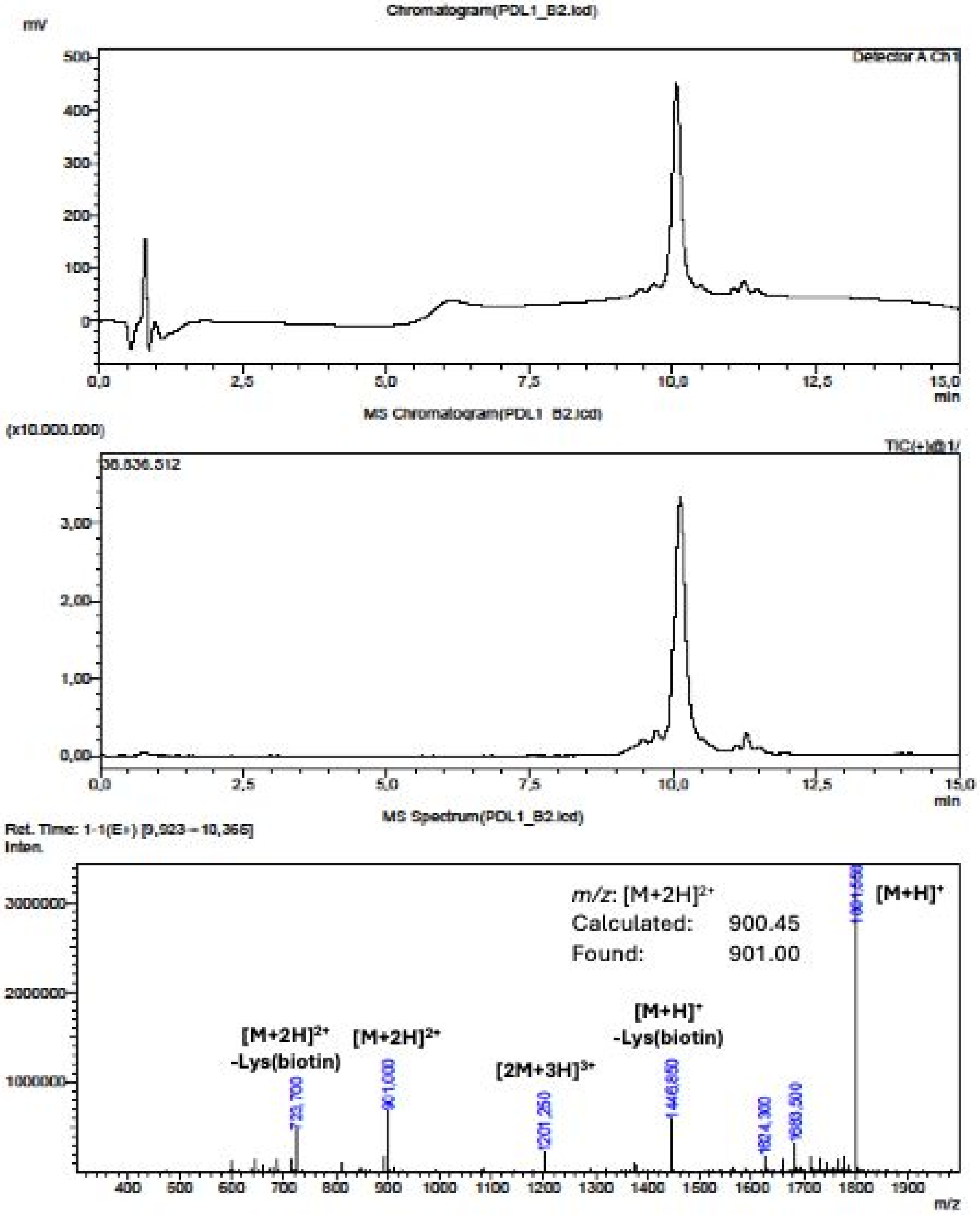

**Figure S44: LC-MS Analysis PDL1_B5**

**PDL1_B5:**
Ac-K(Biotin)-βA-MSGMEVFQRLVEEQRA-$CONH_2$

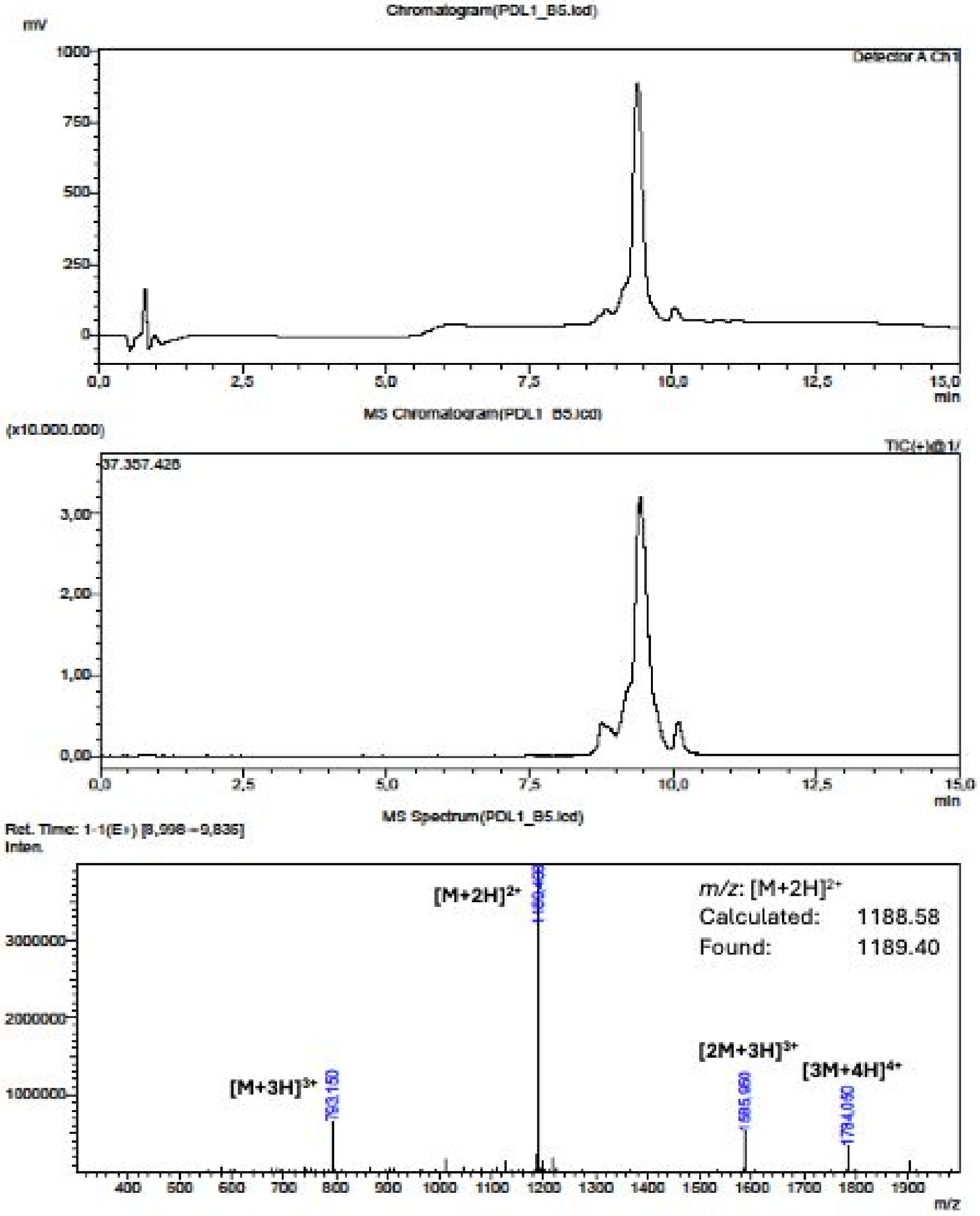

**Figure S45: LC-MS Analysis PDL1_B7**

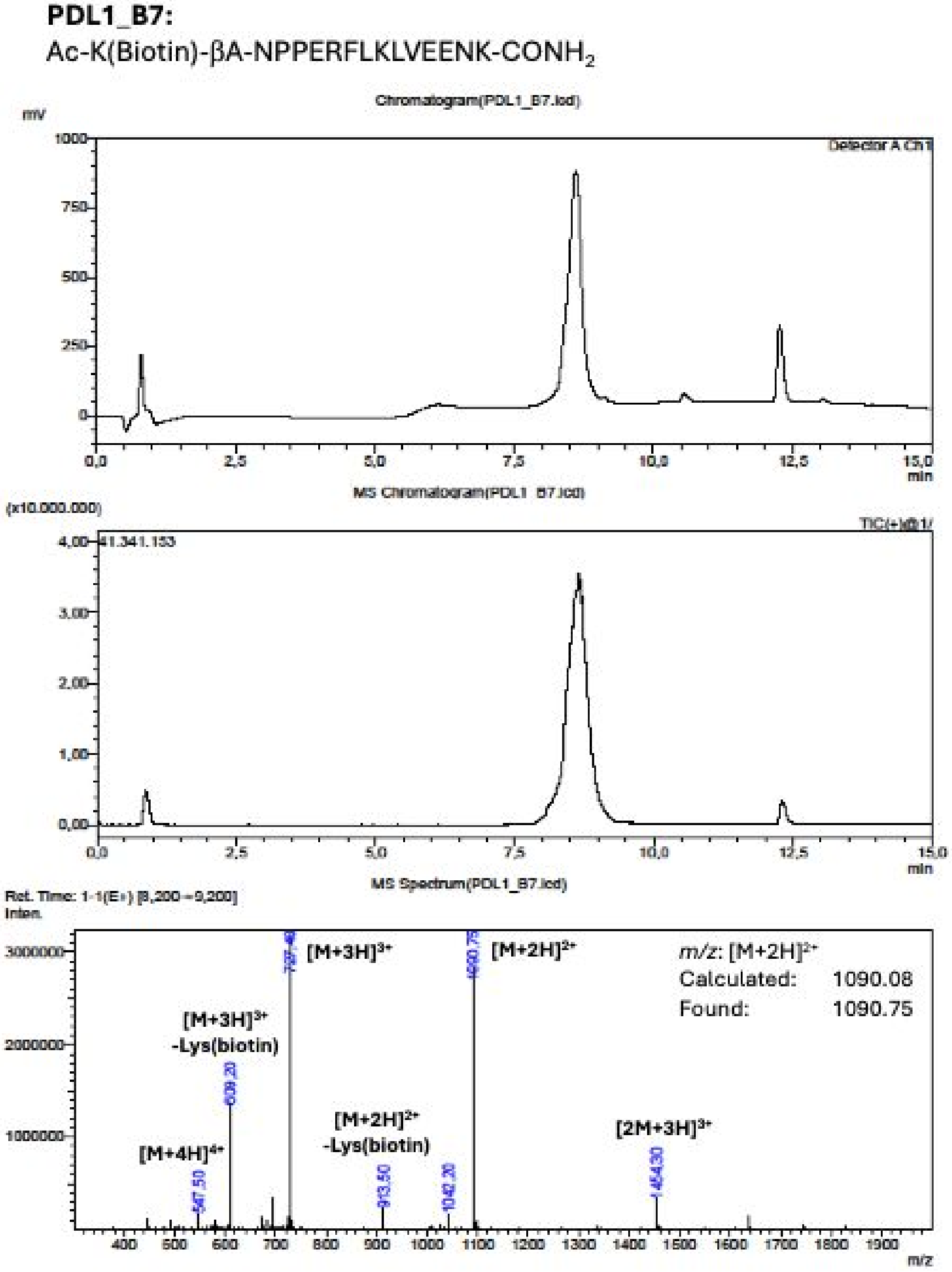

**Figure S46: LC-MS Analysis PDL1_B11**

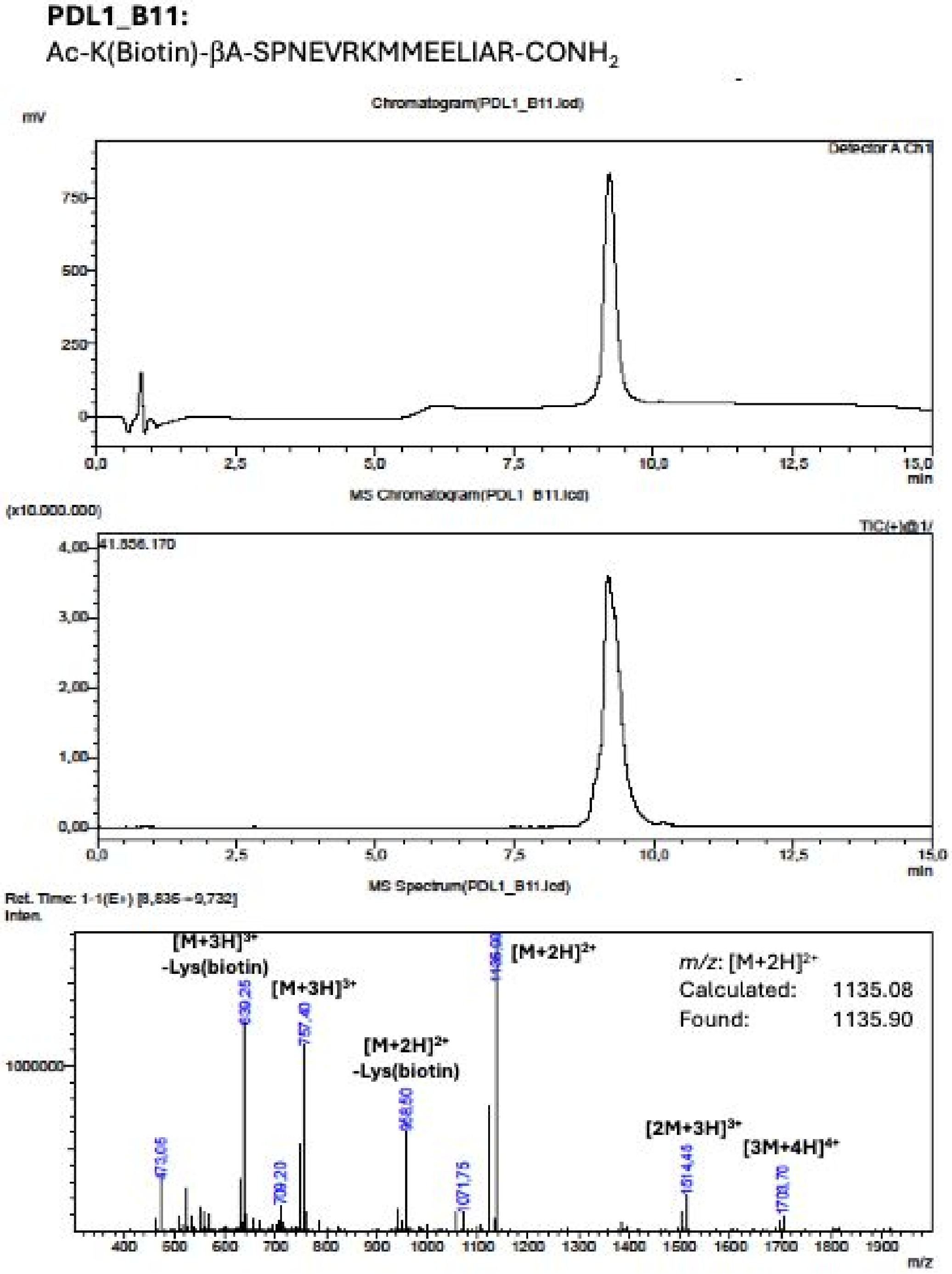

**Figure S47: LC-MS Analysis PDL1_B12**

**PDL1_B12:**

Ac-K(Biotin)-βA-SMEEFQALINR-$CONH_2$

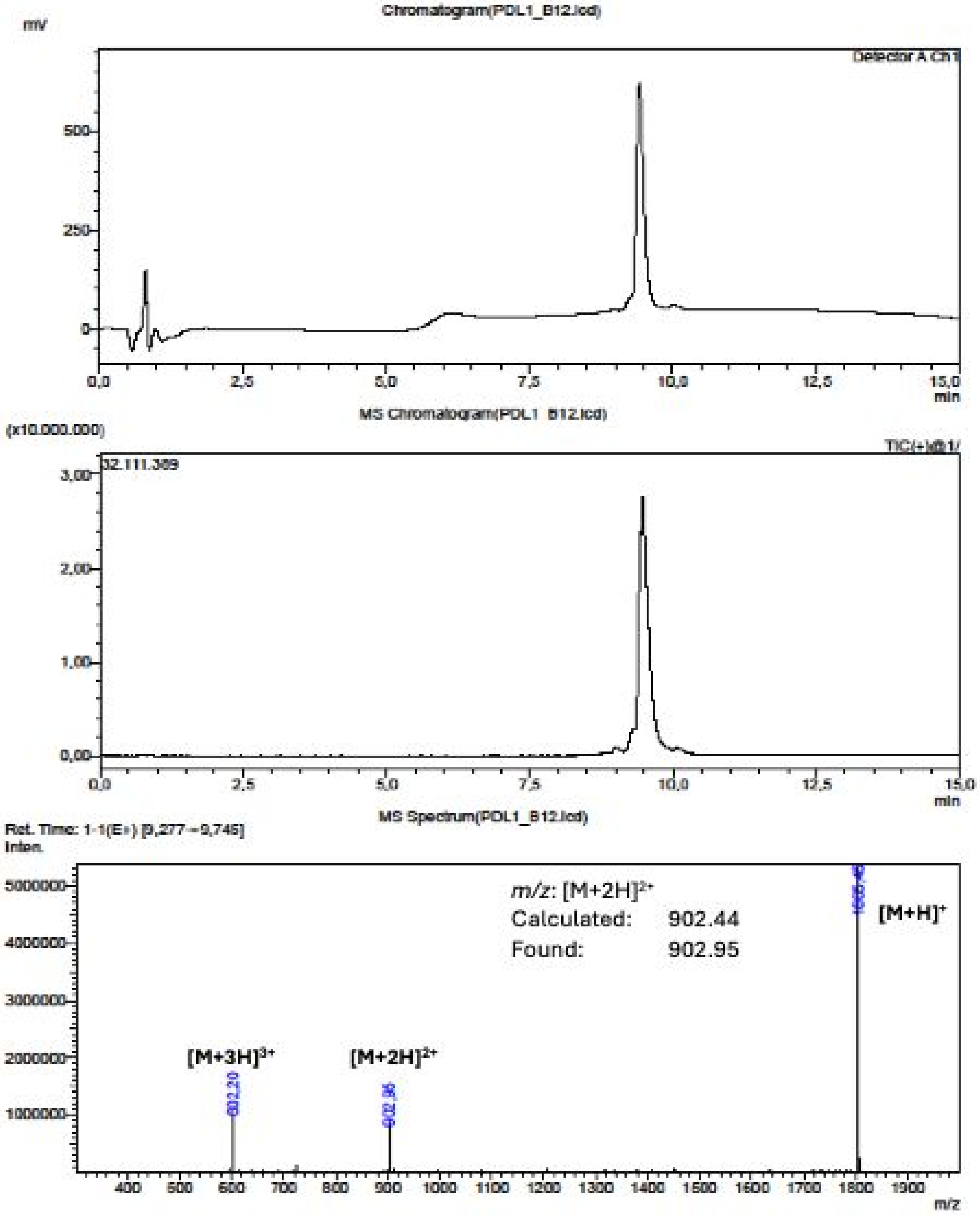

**Figure S48: LC-MS Analysis PD1_B1**

**PD1_B1:**

Ac-K(Biotin)-βA-TDEEGRKKMWEDAYETMKKA-$CONH_2$

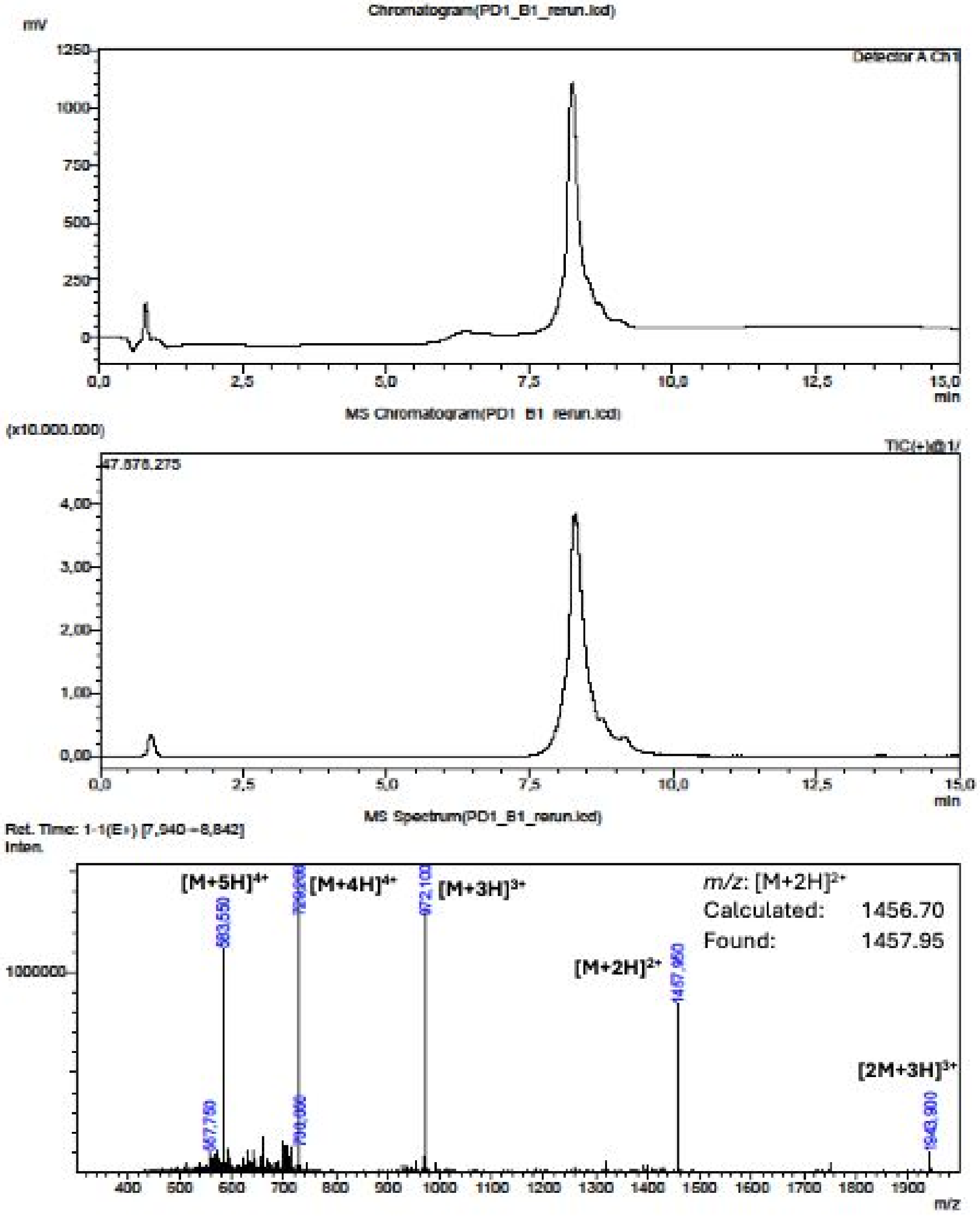

**Figure S49: LC-MS Analysis PD1_B3**

**PD1_B3:**

Ac-K(Biotin)-βA-SPNMEKWLQDAKDDL-$CONH_2$

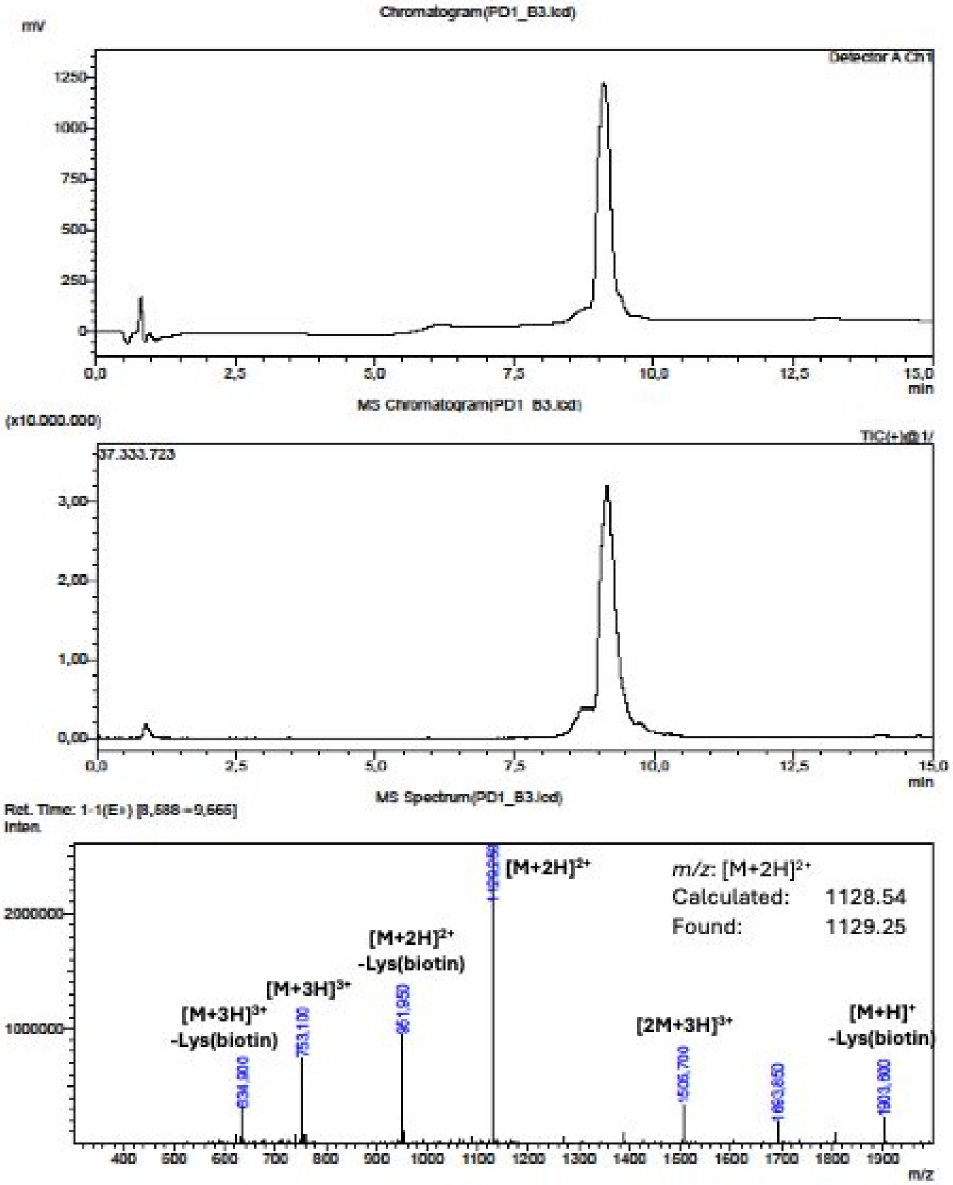

**Figure S50: LC-MS Analysis PD1_B5**

**PD1_B5:**

Ac-K(Biotin)-βA-SPRDAEVEEAKKMLA-$CONH_2$

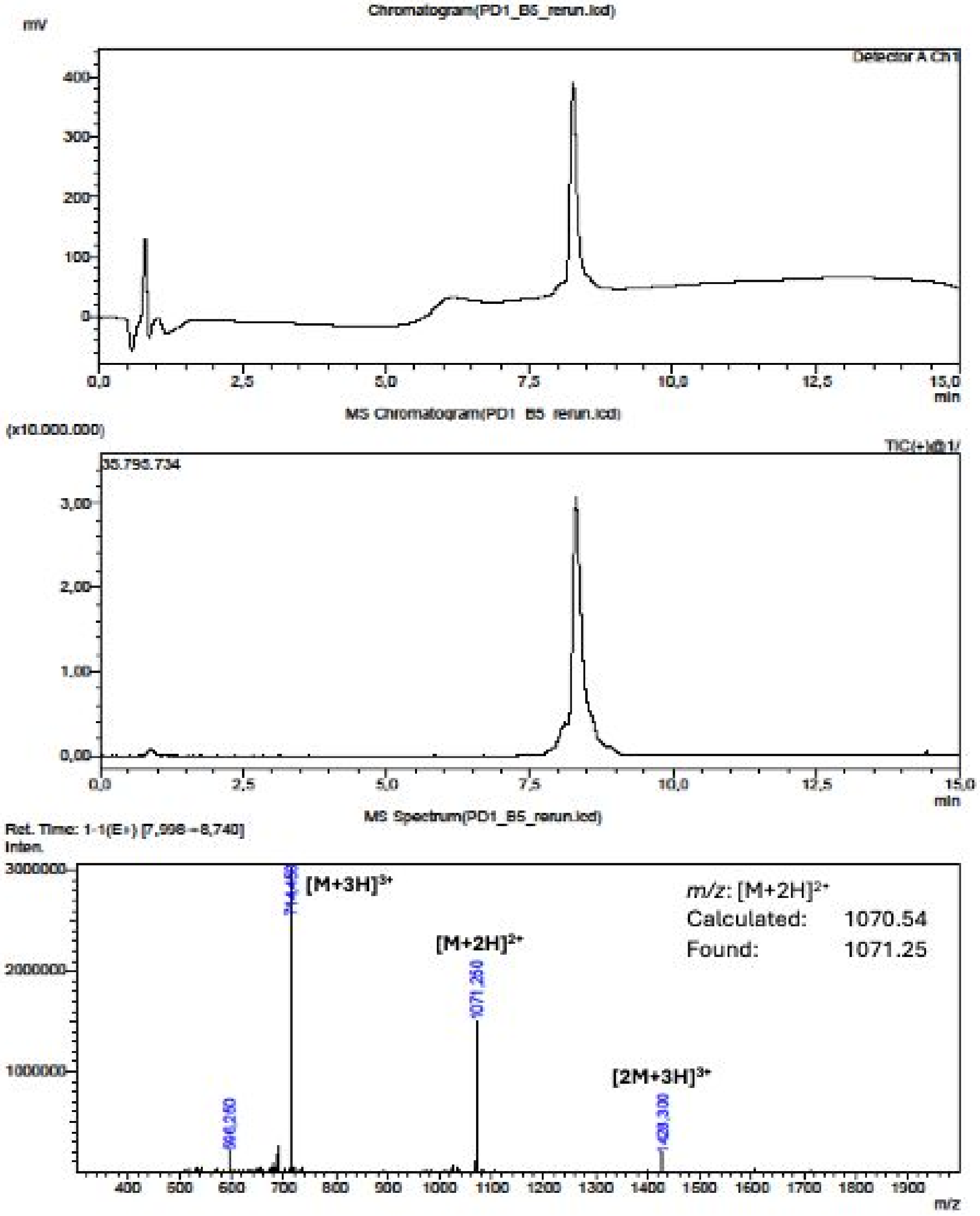

**Figure S51: LC-MS Analysis PD1_B12**

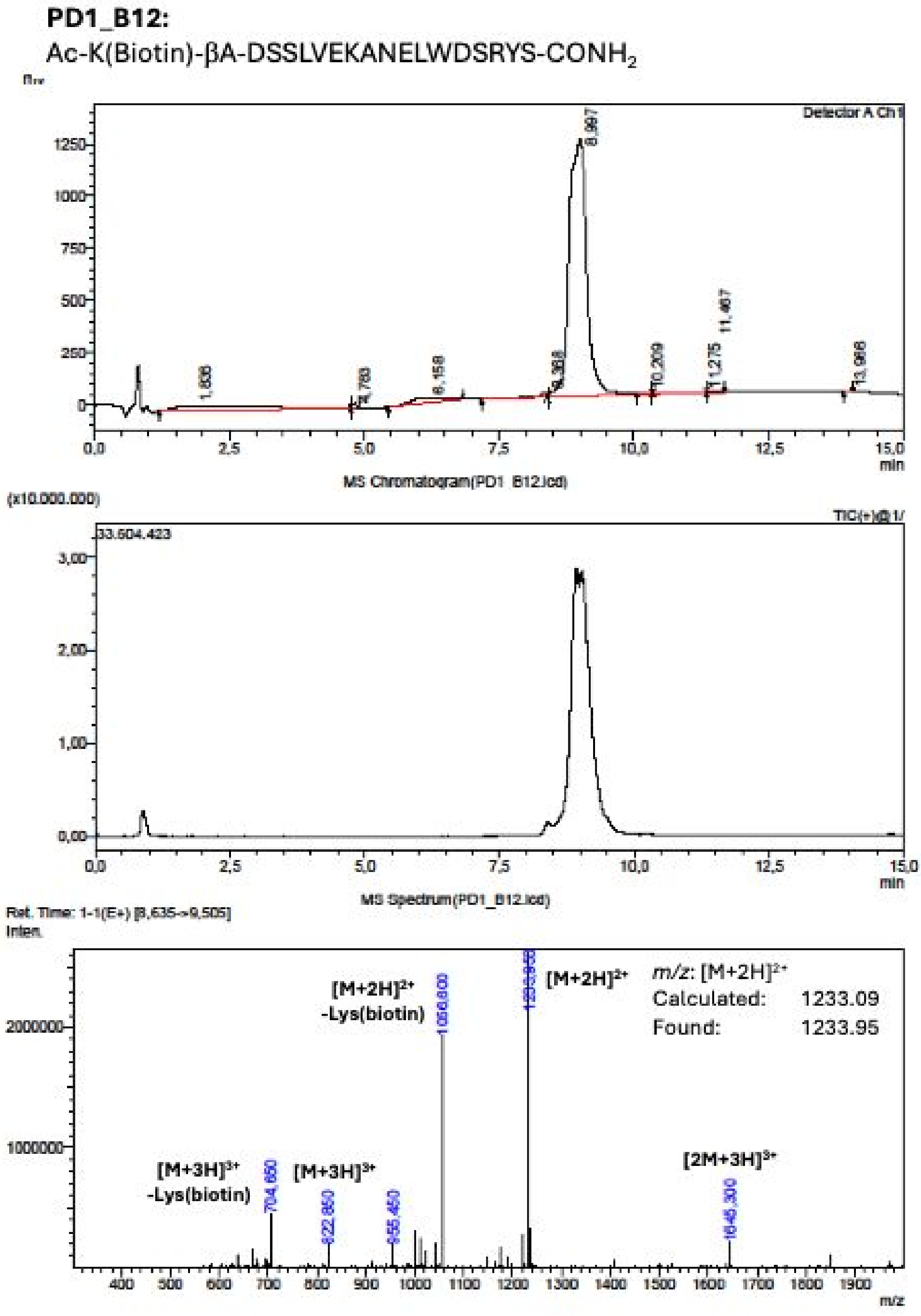

**Figure S52: LC-MS Analysis PD1_B16**

**PD1_B16:**

Ac-K(Biotin)-βA-SISDGLRKDYESLLS-$CONH_2$

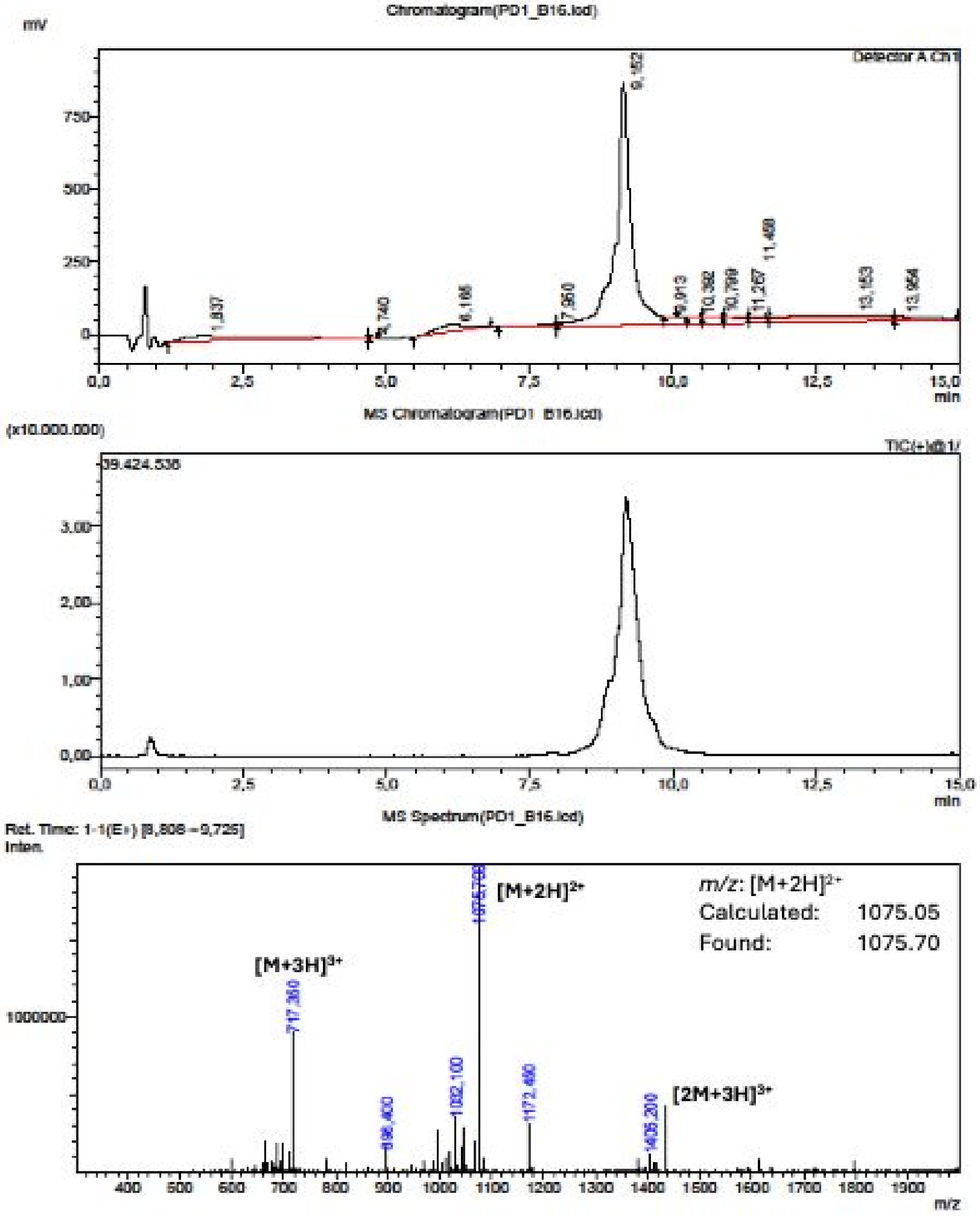

Supplement: Supplementary file 1 [file cb5c00774_si_001.pdf]
